# Immune mechanisms affected by cyclooxygenase inhibition combined with antiviral treatment in calves infected with bovine respiratory syncytial virus

**DOI:** 10.1371/journal.pone.0321642

**Published:** 2025-04-22

**Authors:** Maxim Lebedev, Paul Walsh, John W. Newman, Victoria N. Mutua, Heather A. McEligot, Francisco R. Carvallo Chaigneau, Laurel J. Gershwin

**Affiliations:** 1 Department of Pathology, Microbiology and Immunology, School of Veterinary Medicine, University of California Davis, Davis, California, United States of America; 2 Pediatric Emergency Medicine, Sutter Medical Center Sacramento, Sacramento, California, United States of America; 3 Obesity and Metabolism Research Unit, USDA, ARS, Western Human Nutrition Research Center, Davis, California, United States of AmericaDepartment of Nutrition, University of California, Davis, California, United States of America; 4 Department of Biomedical Sciences & Pathobiology, Virginia-Maryland College of Veterinary Medicine, Virginia Tech., Blacksburg, Virginia, United States of America; Michigan State University, UNITED STATES OF AMERICA

## Abstract

Bovine respiratory syncytial virus (BRSV) infection is a part of the bovine respiratory disease complex. This is one of the most significant problems in both the dairy and beef production sector, inflicting severe economic damage to the industry. BRSV manifests clinically as a respiratory syndrome, affecting both upper and lower respiratory tract, including bronchiolitis with dyspnea and wheezing. It has been shown previously that these symptoms caused by IL-4/IL-13 domination in the immune response are associated with an antibody isotype switch to IgE. Prostaglandin production, such as PGE_2_ is another factor contributing to the pathogenesis of the disease. In this work we demonstrated the effects of ibuprofen and antiviral fusion protein inhibitor (FPI) separately and combined. We showed the synergistic effect of ibuprofen in combination with FPI on antiviral effects and suppression of PGE_2_, resulting in improved cytoplasmic toll-like receptor recognition and humoral immune responses mediated by antimicrobial peptide in lungs. We also demonstrated a Th1/Th2 balance shift towards a Th2 response in lungs and mediastinal lymph nodes, favorable to IL-4/IL-13 responses. This shift may explain the factors contributing to higher viral loads and the lack of histopathological improvement with ibuprofen administered without FPI. Additionally, we demonstrated that endocannabinoids may play a crucial role as natural regulators of the inflammation, adaptive immune response, and resolution of the inflammatory process.

## 1. Introduction

Bovine respiratory-syncytial virus (BRSV) infection in calves is one of the etiological factors of the bovine respiratory disease complex (BRDC) [1]. This condition is caused by the complex of predisposing factors and multiple pathogens [2]. BRDC is common and one of the greatest problems in both the dairy and beef industries. The resultant morbidity and mortality of affected cattle causes severe economic loss. [3,4].

BRSV is a single-stranded negative-sense RNA virus, belonging to the genus Orthopneumovirus and family Pneumoviridae (https://talk.ictvonline.org/taxonomy/). With the high tropism of this virus to epithelial cells of the upper- and lower respiratory tract, manifestation of the disease is often characterized as rhinitis, laryngopharyngitis, tracheo-bronchitis with fever, mucoid nasal discharge, and cough. If the disease progresses to a more severe form and infection involves the lower respiratory tract, it is typically characterized as bronchiolitis with wheezing, tachypnea, cough, fever, and depression [5].

The innate immune response to BRSV is initiated by the recognition of the pathogen-associated molecular patterns by toll-like receptors in epithelial cells, local macrophages and dendritic cells [6]. It has been demonstrated, using human respiratory syncytial virus (HRSV), that TLR2 and TLR6 [7] are involved in the recognition as well as TLR3 [8] and TLR7 [9]. Bovine RSV recognition through TLR3 in γδ T cells activates chemokine and cytokine production responses, including CCL2, CCL3, IL-10, TGF-β [10]. Regulation of cytokines production in lung lesions caused by BRSV includes increased levels of expression of IL-12, IFN-γ, TNF-α, IL-6, IL-18, IL-8, IFN-α and IFN-β [11,12]. BRSV is also able to induce production of IL-1β in infected bovine monocytes and epithelial cells [13].

The adaptive immune response to the virus includes both humoral and cellular immune mechanisms with the cellular immunity as the most important in protection and viral clearance. It has been demonstrated that the humoral immune response to BRSV typically involves IgA and IgG production and often antiviral IgE [14] which plays a crucial role in the pathogenesis of bronchiolitis [15]. Allergic sensitization by viral antigens is a result of the Th2-biased response which occurs at early stages of the infection and is characterized by early onset of IL-4 production (4 days post-inoculation) and antiviral IgE production, which correlated with severity of the disease symptoms [16, 17, 18]. As a result, the adaptive cellular response to BRSV infection is suppressed by early IL-4 production, at least in the initial stages of the infection [14,19]. However, in later stages of the disease CD8+ cytotoxic cells play an important role in virus clearance and establishment of memory [6].

Additionally, increased prostanoid production is another crucial factor in the pathogenesis of the severe BRSV infection as well as human RSV infection. Production of prostaglandin E_2_ (PGE_2_) occurs in early stages of the infection and plays a crucial role in the symptoms and severity of the disease. In later stages, other prostanoids such as Thromboxane B_2_ (TxB_2_) increased in plasma of infected animals [20]. Reduction of prostanoid production using cyclooxygenase (COX) inhibitors can reduce severity of BRSV disease symptoms [21, 22].

In this study we have tested a fusion protein inhibitor GS-561937 (Gilead Sciences) alone and in combination with ibuprofen for the treatment of BRSV. We have previously demonstrated that the anti-inflammatory effect of ibuprofen, despite helping to reduce disease severity, negatively affects infection dynamics, increasing viral loads and viral shedding. In contrast, when combined these agents have apparent synergistic effects reducing disease symptoms, pathology and viral loads, especially when treatment is started on earlier stages of the infection [22, 23, 24]. In this article we further investigate the immune response to the BRSV, how it correlates to the eicosanoid metabolomics and the effect of our therapeutic interventions on these responses.

## 2. Materials and methods

### 2.1. Animals and animal procedures

**Fig 1:**
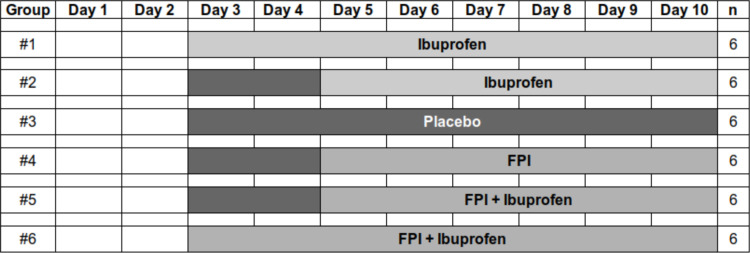
Study design.

Pre-ruminant bovine Holstein calves (*Bos taurus*) were used in this study and all the procedures and experimental conditions were approved by the University of California Davis Institutional Animal Care and Use Committee (authorization number 19313). During the infection period, the calves were monitored closely, with body temperature recorded twice daily and clinical signs assessed daily. Calves exhibiting severe dyspnea and/or an inability to stand or consume milk were designated for humane euthanasia via pentobarbital overdose. For lung lavage procedures, lidocaine was applied intranasally and as a gel on the lavage tube to minimize discomfort. At the conclusion of the experiment, all animals were euthanized with a pentobarbital overdose prior to necropsy. Details of the study design, randomization, interventions, outcomes, handling and detection of the virus have been published elsewhere [22]. Briefly, 36 healthy five to six-week-old outbred pre-ruminant bottle-fed Holstein bull calves were randomly distributed into 6 treatment groups. All animals were infected using individual face masks with nebulizers. Each animal received 5 mL aerosolized infected bovine turbinate cell culture supernatant. The viral infection dose was 3.9×10^5^–9.7×10^5^ PFU. The following treatment groups were created (**Fig 1**): Group 1 – ibuprofen, starting on day 3 post infection (ibp-d3); Group 2 – ibuprofen, starting on day 5 post infection (ibp-d5); Group – 3, placebo; Group 4 – the antiviral fusion protein inhibitor (FPI), starting on day 3 post infection (fpi-d3); Group 5 – FPI and ibuprofen starting on day 5 post infection (fpi+ibp-d5); Group 6 – FPI and ibuprofen starting on day 3 post infection fpi+ibp-d3. GS-561937 (FPI) was administered 600 mg per animal in 30 mL of a mixture of propylene glycol and First Street Snow Cone Syrup (Amerifoods Inc., Los Angeles, CA) once daily.

Ibuprofen was administered three times per day at 10 mg/kg in the form of Advil suspension for children (100 mg/5mL — Pfizer Inc., Madison, NJ). The First Street Snow Cone Syrup without ibuprofen served as an ibuprofen placebo, as well as its 50:50 mix with propylene glycol without FPI for those groups that did not receive FPI. All therapeutics, including placebo, were administered by the oral route individually using catheter-tipped syringes.

Clinical scores, as one of the outcome measurements, were evaluated and points were assigned as described elsewhere [22,25]. Nasal swabs were collected daily for RNA extraction and qRT-PCR was performed to monitor virus shedding as described [22]. On day 10 post infection all animals were euthanized with an intravenous injection of sodium pentobarbital and necropsy was performed. Bronchoalveolar lavage (BAL) was collected 1–2 days before infection and at necropsy, using a sterile foal stomach tube with an internal tube and 3-way stop cock. The tube was advanced to the bifurcation and then three 60 mL volumes of RPMI cell culture medium were injected into the bronchial lumen of the left lung lobe. These samples were then immediately aspirated and placed on ice. Lavage samples for metabolomics and gene expression analysis were stored at -80 °C.

### 2.2. Isolation of peripheral blood mononuclear cells (PBMC)

Blood samples from all animals were collected from the jugular vein using vacutainer and heparin vacutainer tubes to prevent blood coagulation. PBMC were isolated using density gradient centrifugation as follows: 20 mL of blood (undiluted) was layered over 15 mL of Lymphoprep™ (Stemcell Technologies, Vancouver, BC, Canada) that was preloaded in SepMate tube (Stemcell Technologies). The tubes were centrifuged at 1200g for 30 min. The PBMC-containing top layer was transferred to a centrifuge tube and washed using PBS. Isolated PBMC were resuspended in the freezing medium (Fetal bovine serum with 10% DMSO) and frozen using Nalgene Mr. Frosty freezing containers (Thermo Fisher Scientific, Walthem, MA) at -80 °C. After 24 h vials with frozen PBMC were transferred to a storage container with liquid nitrogen.

### 2.3. Flow cytometry

Vials with cells were removed from liquid nitrogen and thawed immediately and quickly in a water bath at 37 °C. The entire content was transferred from the vials to test tubes prefilled with 10 mL of PBS and centrifuged at 350 g for 10 min. Supernatant was removed and cells were washed in 5 ml of PBS (350 g for 5 min). Supernatant was removed and cells were resuspended in 2 ml of working solution of LIVE/DEAD™ Fixable Near IR (780) Viability Kit, for 633 nm excitation (Molecular Probes, Eugene, OR), prepared according to the manufacturer’s protocol, and incubated 30 min at 2–4 °C. After the incubation tubes were centrifuged (350 g for 5 min), supernatant was removed, and cells were washed in 2 mL of PBS with 1% of FCS (PBS-FCS). Next cells were resuspended in PBS-FCS in the volume that was sufficient to load appropriate wells in the 96-well plate. The loaded 96-well plate was centrifuged at 350 g for 2 min and the supernatant was removed. Immediately, the working antibody solutions were added, 50 µL per well (CD4, CD8, CD14, CD25 and CD45RO) to the appropriate wells, as well as PBS-FCS to negative (unstained) control wells. The palate was incubated for 15 min at 4 °C. After the incubation the plate was centrifuged (350 g, 2 min), supernatant removed, and cells washed in 150 µL/well of PBS two times. For γδ TCR staining 50 µL of Zenon-labeled antibody was added to the appropriate wells and incubated for 15 min at 4 °C. γδ TCR-specific antibody was pre labeled using Zenon kit (Thermo Fisher Scientific) according to the manufacturer’s protocol. After the labeling the antibody solution was kept on ice until use (same day, maximum within next 3 hours). After staining with Zenon-prelabeled antibody (for the γδ TSR) cells were washed using 150 µL/well of PBS-FBS two times and fixed with 1% paraformaldehyde (PFA).

For FoxP3 intracellular staining, cells were resuspended in 100 µL/well of 1% PFA in PBS, incubated 10 min at RT (protected from light), then centrifuged down at 350 g, 2min, washed with 150 µL of PBS-FCS one time and permeabilized using 0.1% saponin with 20% normal mouse serum in PBS for 1.5 h at 4 °C. Working dilution of FoxP3 antibody in permeabilization buffer (0.1% saponin in PBS) without normal mouse serum was prepared and added to permeabilized cells (50 µL/well), sealed and incubated for 1 hour at 4 °C. After the incubation cells were washed in 150 µL of permeabilization buffer (0.1% saponin in PBS) and another wash was performed in 150 µL of PBS-FCS and cells resuspended in 1% PFA in PBS. Cells were refrigerated (2–4 °C) until flow cytometry procedure. Flow cytometry was performed using the CytoFLEX instrument (Beckman Coulter, Brea, CA), data analyzed using FlowJo software (BD Biosciences, Franklin Lakes, NJ). The collected flow cytometry data parameter was the frequency of the cell population of interest, expressed as a percentage of the parent population. The gating strategy involved the elimination of dead cells using live/dead differentiation staining with the cell viability staining kit mentioned above. Next, single cells were selected using an FSC-A vs. FSC-H scatter plot. Monocytes were distinguished from lymphocytes based on cell size using an FSC vs. SSC plot and further identified by CD14+ staining. Lymphocytes gated on the FSC vs. SSC plot were subsequently subdivided based on CD4+, CD8+, and TCR γ-chain-specific staining for γδ T cells. Further differentiation of T cell subsets was achieved through the detection of additional markers, such as CD25 for activated cells, CD45RO for memory phenotype, and FoxP3 for regulatory T cells. Representative gating is shown in Supplementary S1 File. To assess relative changes in each cell subpopulation throughout the experiment, values were normalized to baseline. Specifically, the ratios of frequencies under experimental conditions to the pre-infection time point (baseline) were calculated for each animal, and the average normalized values were determined for each treatment group and experimental time point.

Antibodies used in this study are listed in Table 1.

**Table 1:** Antibodies used for flow cytometry.

Antibody	Clone	Fluorophore	Manufacturer
Human anti-bovine FoxP3	7627	Alexa Fluor® 647	Bio-Rad, Hercules, CA
Mouse anti-bovine CD4	CC8	R-PE	Bio-Rad
Mouse anti-bovine CD8	CC63	R-PE	Bio-Rad
Mouse anti-bovine CD25	IL-A111	FITC	Bio-Rad
Mouse anti-bovine CD14	CC-G33	FITC	Bio-Rad
Mouse anti-bovine CD45RO	IL-A116	Qdot 655 SiteClick™ (Thermo Fisher Scientific)	Bio-Rad,
Mouse anti-bovine TCR1-N7 γ-chain-specific IgG1	CACTB81A	R-PE Zenon^TM^ (Thermo Fisher Scientific)	Washington State University Monoclonal Antibody Center

Qdot 655 SiteClick™ Antibody Labeling Kit and R-PE ZenonTM Mouse IgG1 Labeling Kit were used according to the manufacturer’s manuals.

### 2.4. Tissue samples collection and RNA extraction

Bronchial lymph nodes (directly adjacent to large bronchi) and caudal mediastinal lymph nodes (in the mediastinum, located near esophagus and aorta) were collected at necropsy on day 10 after infection, immediately flash-frozen in liquid nitrogen, and stored at -80 °C. A thin slice (30–40 mg) of each frozen lymph node tissue sample was taken using a razor blade and immediately placed into 0.6 mL of buffer RLT (Qiagen, Hilden, Germany) on the surface of a sterile Petri dish to start lysis. Once in the lysis buffer, the slice of tissue was chopped with the razor blade to multiple smaller pieces then placed into a 1.5 mL Eppendorf tube and vortexed for better homogenization. As a final homogenization step, samples were centrifuged through the QIA shredder spin column (Qiagen). Centrifugate was diluted 1/1 with 70% alcohol and RNA extracted using RNeasy Mini Kit (Qiagen). Residual genomic DNA was digested on-column by RNAse-Free DNAse Set (Qiagen) After elution, RNA was tested for concentration and contamination using NanoDrop 2000c spectrophotometer (Thermo Fisher Scientific) and tested for RNA integrity, using Bioanalyzer 2100 (Agilent, Santa Clara, CA USA). RNA quality control data is provided in the supplementary S2 File. After extraction and quality control, RNA was stored at -80 °C.

### 2.5. RNA sequencing

Library generation and sequencing was performed in the DNA technology & Expression Analysis Core Laboratory at the University of California Davis as described previously [24]. Briefly, Tag-Seq libraries were prepared using the QuantSeq FWD kit (Lexogen, Vienna, Austria) for multiplexed sequencing. The fragment size distribution of the libraries was verified by Bioanalyzer 2100 (Agilent Technologies, Santa Clara, CA). The libraries were quantified by fluorometry on a Qubit instrument (Life Technologies, Carlsbad, CA), and pooled in equimolar ratios. Forty-eight libraries were sequenced per lane on a HiSeq 4000 sequencer (Illumina, San Diego, CA) with single-end 100 bp reads. The sequencing generated more than 3 million reads per library.

### 2.6. Analysis of RNA sequencing data

Analysis of the sequencing data and differential gene expression was performed by UC Davis Bioinformatics Core.

PhiX screening, adapter trimming and quality trimming were conducted using HTStream, version 1.0.0 (https://github.com/s4hts/HTStream). Alignment to Bos_taurus.ARS-UCD1.2 and read counting were conducted using STAR, version 2.7.0f [26]. Parameters used are provided as supplementary S3 File and S4 File.

Differential expression analyses were conducted using limma-voom [27]. The model fitted included effects for sample type, treatment, the interaction between sample type and treatment, sequencing pool, and RNA isolation date. Standard errors of log fold changes were adjusted for within-animal correlations. Genes reported as significant are those with a false discovery rate adjusted p-value less than 0.05. Gene ontology enrichment analysis of the whole transcriptome was conducted using Kolmogorov-Smirnov test as implemented in the Bioconductor package topGO, version 2.30.1.

### 2.7. Lipid mediator and ibuprofen extraction and analysis

Lipid mediators of immune function were quantified using liquid chromatography with tandem mass spectrometry (LC-MS/MS) in bronchial and mediastinal lymph nodes and broncho-alveolar lavage fluids. Measured mediators included oxylipins derived from cyclooxygenase, lipoxygenase, and cytochrome P450 dependent metabolism, as well as the endocannabinoid and endocannabinoid-like monoacylglycerols and acylethanolamides. All samples were extracted in the presence of isotopically labeled surrogates and antioxidants. For lymph tissue, ~30–50mg of frozen tissue was thawed on wet ice and enriched with surrogates in 5 µL of methanol and 5 µL of 0.1 mg/mL butylated hydroxytoluene/EDTA anti-oxidant solution and homogenized with an additional 200 µL of 1:1 methanol:acetonitrile at 1350 RPM on a Genogrinder 2010 (SPEX Sample Prep, Metuchen, NJ) with 3mm stainless steel balls for 5 min. Sample supernatants are then filtered by centrifugation for 2 min through a 0.2 µm PVDF membrane 400 µL 96-well microfilter plate (Agilent Technologies), with eluents collected in 0.45 mL Nunc polypropylene microplates (ThermoFisher Scientific). For broncho-alveolar lavage fluids, 0.25 to 1mL aliquots were thawed on wet ice and transferred to a 2mL polypropylene microtiter plate maintained on wet ice containing isotopically labeled surrogates and the EDTA/BHT anti-oxidant solution, gently mixed and up-diluted with a 30% methanol in water. Oxylipin free acids were then isolated from the chilled samples with a 1 cc 10 mg Oasis HLB solid phase extraction 96-well plates (Waters Corp Inc., Milford, MA). The SPE wells are pre-washed with ethyl acetate and methanol and conditioned with 2 mL 0.1%AA/5%MeOH. The diluted samples are then loaded and extracted by gravity and air dried 20 min with light vacuum. The targets are eluted with 0.5 mL 1.0% acetic acid in methanol, followed by 1.0 mL ethyl acetate, and collected in deep-well polypropylene 96-well plates containing 10 µL of 20% glycerol in methanol. Solvents are removed under vacuum, residues are reconstituted in 100 µL 100nM 1-cyclohexyl-3-ureido dodecanoic acid internal standard solution, chilled, and filtered by centrifugation at 0.2 µm as described above. Filtered samples are mat-capped and stored at -20 °C until analysis LC-MS/MS.

Inflammatory mediators including oxylipins and endocannabinoids were quantified along with ibuprofen against authentic calibration standards by electrospray ionization LC-MS/MS using previously reported methods [28]. Briefly, residues were separated on a 2.1 x 150mm 0.17µm BEH C18 column (Waters Corp) and detected on an API 6500 QTRAP (Sciex, Redwood City, CA) with positive/negative switching using scheduled multi-reaction monitoring. Analytes are generally quantified against 7–9 point calibration curves.

Calibrants and internal standards were purchased from Cayman Chemical (Ann Arbor, MI), Medical Isotopes (Pelham, NH), Avanti Polar Lipids Inc. (Alabaster, AL), or Larodan Fine Lipids (Malmo, Sweden). Data is processed with AB Sciex MultiQuant v. 3.2.

### 2.8. Metabolomics-transcriptomics integration

As a first step in data integration, weighted correlation network analysis (WGCNA) was performed on the transcriptomics data to identify clusters of co-expressed genes [29]. These clusters were then correlated with the metabolomics data. For the WGCNA analysis, genes with fewer than 4 counts per million reads in all samples were filtered prior to analysis. A soft thresholding power of 6 and a signed network were used in WGCNA. Gene Ontology (GO) enrichment, using Fisher’s Exact Test and the biological process ontology, was performed to characterize the genes in each module. The Pearson correlation was performed between the indicated module and metabolite. Due to the large number of tests conducted, only very small p-values (around 0.0001 or less) were used as statistically significant.

### 2.9. Functional enrichment analysis of differentially expressed genes and visualization of functional networks

The analysis was performed using Cytoscape/ClueGo version 2.5.9 [30]. Functional enrichment analysis was conducted using a two-sided hypergeometric test with Bonferroni step down correction. Latest “GO Biological Process”, “GO Immune System Process” were used. GO levels were set in the interval from 3 to 8, minimum gene threshold of 2 genes per GO term. The Kappa score threshold of 0.4 was set to determine term-term interactions and visualization of term connections as a network. ClueGo 2.5.9 was also used for visualization of networks, comparative analysis and sorting of whole transcriptome enrichment results using predefined terms from topGO analyses.

### 2.10. Quantitative polymerase chain reaction

IL-17, IL-4 and IFN-γ gene expression in bronchial lymph nodes was performed in UC Davis Real-time PCR Research and Diagnostics Core Facility, using the following procedures.

*Nucleic acid extraction -* Approximately 5 mg of tissue was added to a 96-well deep-well grinding block (Greiner Bio-One, Monroe, North Carolina, USA) with 600 µL of ATL Buffer, 60 µL of Proteinase K (Qiagen, Valencia, California, USA), and two stainless-steel beads (Thermo Fisher Scientific). Samples were homogenized using a 2010 Geno/Grinder homogenizer (SPEX SamplePrep, Metuchen, New Jersey, USA) at 1,750 rpm for 2.5 min. Lysate was incubated for 15 min at 56 °C. 200 µl of lysate was removed and used for total nucleic acid (TNA) extraction. TNA extraction was performed on a semi-automated extraction system (QIAamp 96 DNA QIAcube HT Kit, QIAcube; Qiagen) according to manufacturer’s instructions and eluted in 100 µL of diethylpyrocarbonate (DEPC)-treated water.

c*DNA synthesis* - Complementary DNA (cDNA) was synthesized from 10 uL of extracted TNA using the QuantiTect Reverse Transcription Kit (Qiagen) following the manufacturer’s recommendations. To ensure all DNA was digested, a background check was performed on a subset of six samples. This included running 1 µL of digested TNA for bovine GAPDH. A negative bGAPDH signal indicated that DNA was successfully eliminated and not carried over into cDNA synthesis.

*qPCR -* Each qPCR reaction contained 20X primers and a probe for the qPCR assay with a final concentration of 400 nM for each primer and 80 nM for the probe. Commercially available PCR master mix (TaqMan TM Universal PCR Master Mix, ThermoFisher Scientific) containing 10 mM Tris-HCl (pH 8.3), 50 mM KCl, 5 mM MgCl 2, 2.5 mM deoxynucleotide triphosphates, 0.625 U AmpliTaq Gold DNA polymerase per reaction, 0.25 U AmpErase UNG per reaction and 5 µL of cDNA were added to the PCR reaction in a final volume of 12 µL. The samples, in addition to positive and negative PCR controls, were placed in a 384-well plate. Amplification was performed under the following conditions on a QuantStudio Q7 Pro (Applied Biosystems): 2 min at 50 °C to, 10 min at 95 °C, followed by 40 cycles of 95 °C for 15 s and 60 °C for 1 min. Fluorescent signals were collected during the annealing temperature and the quantitative cycle (Cq) was calculated and exported with a threshold of 0.2 and a baseline of 3–10.

*Real-time PCR Assay Development -* The gene profile consisted of three genes of interest (GOI) and one reference gene. GOIs included bovine IL-17 (GenBank sequence accession # AF412040, efficiency of 91.10%), IL4 (M77120, 97.30%), and IFNg (ThermoFisher Scientific, Bt032123723_m1, 93.43%), while the reference gene was GAPDH (NM_001034034, 110.20%). Primer sequences, probes and other details are provided in supplementary S5 File. All assays were validated for efficiency and sensitivity by running a 10-fold standard curve in triplicate of serial dilutions made from a biological control.

Results of the group 3 (placebo) were used as a reference sample to determine the ΔΔCq in other treatment groups.

### 2.11. Data handling and analysis

Microsoft Excel was used to manage flow cytometry data spreadsheets, calculations, to generate graphs and analysis of variance with the Tukey post-hoc test. JMP Pro 15 statistical software was used for analysis of correlations between ibuprofen levels and gene expression as well as graphical representation of the results. Methods of transcriptomics and metabolomics analysis are described above, in corresponding sections.

## 3. Results

### 3.1. Analysis of peripheral blood mononuclear cell populations

Changes in peripheral mononuclear cell populations were determined every two days by detection of frequencies of certain cell populations based on the staining for specific surface- and intracellular markers. Ratios of frequencies at each experimental time point vs baseline levels were calculated (raw data for the frequencies and ratios of all cell populations and time points are provided in Supplementary S6 File). Statistically significant differences among experimental groups in every PBMC subset at each time point were not detected, including differences between placebo and all experimental groups However, changes between time points within some cell populations were observed in certain experimental groups.

**Fig 2:**
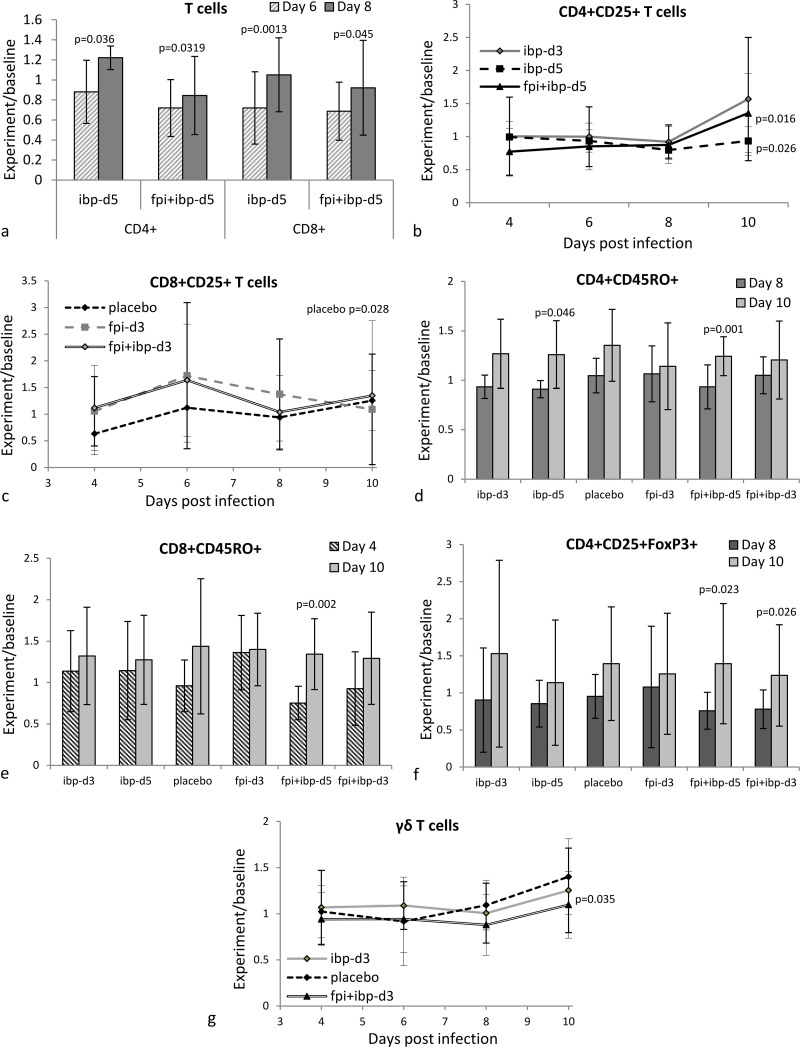
Comparison of treatment/baseline ratios between PBMC populations. **Only results with statistically significant changes are demonstrated.** P-values indicate difference between day 6 and day 8 in (a), day 8 and day 10 in (b) and (c), (d), (f), (g) an day 4 and day 10 in (e). Error bars = standard deviation (n=5). Graphs representing changes in baseline-normalized levels of all cell populations at all time points are presented in supplementary S7 File.

Pattern changes in major populations of lymphocytes and in monocytes were observed. Elevation in experiment/baseline ratios of both CD4+ and CD8+ cells was determined in most of the treatment groups with statistically significant increases on day 8 if compared with day 6 (previous time point) in animals of group ibp-d5 and group fpi+ibp-d5 (Fig 2a). This ratio was the highest level of these populations over the entire course of our observation and there was a tendency for the reduction of this ratio on day 10, but it was not statistically significant (data not shown). Treatment/baseline ratios of monocytes demonstrated similar trends as lymphocytes, but all the differences among time points were not statistically significant (data not shown).

Experiment-to-baseline ratios of activated Th cells (CD4+CD25+) did not change over the course of observation, and were not different from the baseline, except for the day 10 where the ratios increased in the presence of ibuprofen (i.e., ibp-d3, ibp-d5 and fpi+ibp-d5). The increase was statistically significant for groups ibp-d5 and ibp+fpi-d5 where ibuprofen treatment began on day 5. (Fig 2b).

Activated CD8+ T cells (CD8+CD25+) demonstrated different patterns, but due to high data variability, most of the changes were not statistically significant. However, mean experiment-to-baseline ratios demonstrated different patterns. In the placebo group the ratio tended to increase, especially after day 4 and at day 10 (p =0.028). All groups demonstrated reduction of activated CD8+ cells experiment/baseline ratios between day 6 and day 8 (**Fig 2c**). Groups ibp-d3, ibp-d5 and fpi+ibp-d5 demonstrated reduction of activated CD8+ cells from day 4 to day 8 interval with increase at day10. This increase was also observed in most of the experimental groups.

Normalized frequencies of T-helper cells with memory phenotype (CD4+CD45RO+) in most groups (except group fpi-d3) were elevated at day 10, compared to day 4; this was a statistically significant increase in group ibp-d5 and group fpi+ibp-d5 (Fig 2d). Sufficient changes in the cytotoxic T cell population with memory phenotype (CD8+CD45RO+) were not detected at certain time points, but gradual elevation of this subset during the entire observation period was observed (Fig 2e) with statistically significant differences between day 4 and day 10 in group fpi-ibp-d5.

Experiment/baseline ratios elevation in the T cell population with regulatory phenotype (T-reg, CD4+CD25+FoxP3+) observed only on day 10 (**Fig 2f**) with a statistically significant increase of the levels in groups fpi+ibp-d5 and fpi+ibp-d3. In the placebo group a relative increase of this population was observed on day 8 (p =0.034, data not shown) and the elevation was also present on day 10.

γδ T cells demonstrated similar changes to ones of the T-reg cells at least in 3 experimental groups (ibp-d3, placebo and fpi+ibp-d3) whose baseline-normalized frequencies were elevated on day 10 (Fig 2g). A statistically significant increase was observed only in group ibp-d3. Similarly to T-regs, γδ T cells in the placebo group started increasing earlier (on day 8) than in other experimental groups.

### 3.2. Clusters of co-expressed genes correlate with some eicosanoid levels in bronchoalveolar lavage.

**Fig 3:**
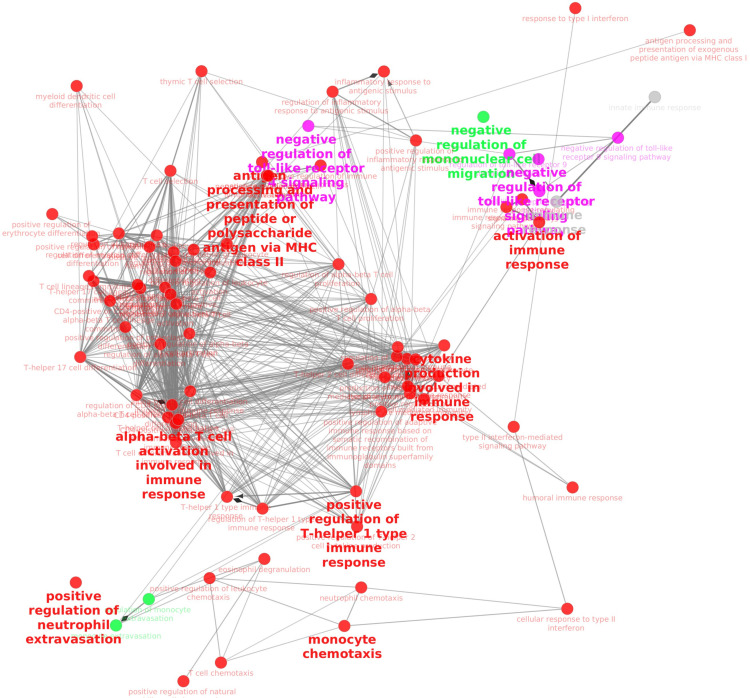
Network of co-expressed gene clusters that correlated with oxylipins in BAL.

Seventeen clusters (WGCNA transcriptomics modules) of co-expressed genes were identified in bronchoalveolar lavage and 19 eicosanoids and ibuprofen were detected and quantified by mass spectrometry. Pearson correlation data between these clusters and eicosanoid levels (supplementary S1 Fig) demonstrated only a few spots with correlation that can be considered as statistically significant (p ≤0.0001 after Bonferroni correction for the number of tests). Thromboxane B2 (TxB2) was positively correlated (ρ = 0.49; p = 0.00002) with a module that includes major groups of genes responsible for regulation of αβ T cell activation, regulation of adaptive immune response and macrophage activation involvement in the immune response. The same gene cluster was negatively correlated (ρ = -0.58, p = 2E-07) with pinellic acid (9,12,13-TriHOME). GO enrichment with subsequent network analysis of enriched GO terms (**Fig 3, red nodes**) demonstrated that this cluster of co-expressed genes is the most abundant among the other 3 clusters that were compared and that GO terms were grouped into similar functional groups, such as “Alpha-beta T cell activation involved in the immune response”, “Positive regulation of T-helper 1 type immune response” and “Antigen processing and presentation of peptide or polysaccharide antigen via MHC class II”. These groups are strongly interconnected, but this cluster also included other groups that were relatively distant and had no direct connections with them, such as “Monocyte chemotaxis” and “positive regulation of neutrophil extravasation” (this group has no connections with others within this cluster of genes).

The following colors were assigned to clusters of co-expressed genes (named by leading GO terms on supplementary S1 Fig): Red – Regulation of alpha-beta T cell activation; Blue – Ribosomal large subunit biogenesis; Purple – Positive regulation of megakaryocyte differentiation; Green – Antimicrobial humoral immune response mediated by antimicrobial peptide.

Another module was positively correlated with prostaglandin E2 (ρ = 0.6, p = 9E-08) (supplementary S1 Fig) and included two groups of genes: Positive regulation of megakaryocyte differentiation and negative regulation of viral-induced cytoplasmic pattern recognition receptor signaling pathway (both terms are statistically significant). This co-expressed gene cluster was also negatively correlated (ρ = -0.45, p = 0.0001) with ibuprofen concentration and Keto-prostaglandin F1-alpha (ρ = -0.39, p = 9E-04). Network analysis of enriched GO terms (Fig 3**, purple nodes**) demonstrated that this cluster includes two major groups: “Negative regulation of toll-like receptor 4 signaling pathway” “Negative regulation of toll-like receptor signaling pathway”.

As for the prostaglandin E2, it was also negatively correlated (ρ = -0.5, p = 0.00001) with a gene cluster that did not contain any immune function GO terms, but was related to ribosomal biogenesis, assembly, formation of cytoplasmic translation complexes and cytoplasmic translation. Interestingly, this gene cluster also positively correlated with ibuprofen concentration (ρ = 0.49, p = 0.00002). Network analysis using “GO Biological Process” instead of only “GO Immune Process” (supplementary S2 Fig) terms showed that “Positive regulation of cyclic-nucleotide phosphodiesterase activity” was interconnected with other clusters.

Another negative correlation (ρ =-0.46, p =8E-05) of prostaglandin E2 was observed with the gene cluster that included groups responsible for “antimicrobial humoral immune response mediated by antimicrobial peptide”, “positive regulation of mast cell degranulation”, T cell activation and proliferation. Network analysis (**Fig 3, green nodes**) revealed only one leading GO term – “Negative regulation of mononuclear cell migration” without connections with other groups or clusters.

### 3.3. Clusters of coexpressed genes have positive or negative correlation with eicosanoids in regional lymph nodes

***Bronchial lymph nodes.*** WGCNA analysis of bronchial lymph nodes tissue revealed 18 clusters of co-expressed genes. A total of 38 lipid mediators and ibuprofen were detected and quantified in this tissue. Multiple occurrences of statistically significant correlation between WGCNA modules and metabolites were detected (supplementary S3 Fig).

**Fig 4:**
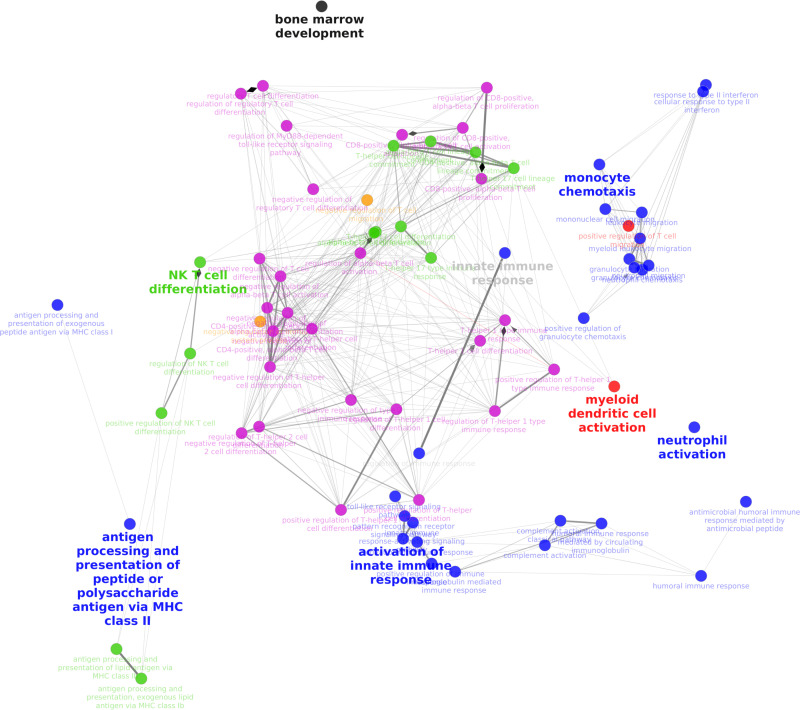
Network of select co-expressed gene clusters in BLN demonstrated correlation with some oxylipins. The following colors were assigned to clusters of co-expressed genes (named by leading GO terms on supplementary S3 Fig): Red – Regulation of B cell differentiation; Blue – Th1 cell cytokine production; Brown – Neutrophil extravasation; Black – T cell costimulation; Green – Negative regulation of T cell receptor signaling pathway; Purple – Alpha-beta t cell differentiation.

13-Hydroxyoctadecadienoic acid (13-HODE) had relatively high level of correlation with two adjacent to each other modules containing the following groups of genes responsible for “Regulation of B cell differentiation”, “Regulation of leukocyte mediated cytotoxicity”, “Negative regulation of leukocyte activation” (ρ = 0.68, p = 6E-08) (Fig 4**, red nodes**). The second module included (Fig 4**, blue nodes**): “Th1 cell cytokine production”, “Pattern recognition receptor signaling pathway”, “Myeloid cell activation involved in immune response” and “Regulation of monocyte chemotaxis” (ρ = 0.61, p = 9E-05). This cluster of co-expressed genes is involved in multiple immune functions (Fig 4) and divided into 3 major relatively distinct groups, including “Antigen processing and presentation of peptide or polysaccharide antigen via MHC class II”, “Activation of innate immune response” and “Monocyte chemotaxis”. Additionally, both of these clusters together were positively correlated with several other metabolites, and the cluster with the major group of genes responsible for Th1 cell cytokine production had higher correlation (that was also statistically significant) with the following metabolites: 9-OxoODE (9-KODE) (ρ = 0.61, p = 9E-05), 15(16)-epoxy-9Z,12Z-octadecadienoic acid (15(16)-EpODE) (ρ = 0.63, p = 3e-05); as well as negative correlation (ρ = -0.60 to -0.71, p ≤0.0001) for both adjacent clusters with homo-gamma-linolenoyl ethanolamide (DGLEA), anandamide (AEA), adrenoyl-ethanolamine (DEA) and N-arachidonylglycine (NA-Gly).

Another cluster that had multiple positive and negative correlations with lipid mediators contained the following major group terms (Fig 4**, brown nodes**): “Neutrophil extravasation”, “Lymphocyte differentiation”, “Myeloid cell activation involved in immune response”. This cluster had statistically significant positive correlation with 12,13- and 9,10- regioisomeric diols of linoleic acid, namely the 12,13-dihydroxy-9Z-octadecenoic acid (12,13-DiHOME; i.e., isoleukotoxin diol) (ρ = 0.68, p = 4E-06) and 9,10-DiHOME (i.e., leukotoxin diol; ρ = 0.65, p =2 E-05). This cluster was negatively correlated with 15-HETE (ρ = -0.69, p = 4E-06). Generally, this cluster also negatively correlated with prostaglandins and had the highest positive correlation (0.4) with ibuprofen levels, p-values did not reach significance after multiple comparison corrections.

Another cluster (adjacent to the previous one) and containing the following major group terms (Fig 4**, black node**): “T cell co-stimulation”, “Positive regulation of MDA-5 signaling pathway”; “Myeloid dendritic cell cytokine production”, positively correlated with 15,16-DiHODE (ρ = 0.63, p = 4E-05), 13-hydroxyoctadecadienoic acid (13-HODE; ρ = 0.65, p = 2E-05) and (15(16)-EpODE; ρ = 0.64, p = 2e-05).

The last co-expressed gene cluster (Fig 4**, green nodes**) that demonstrated statistically significant correlation with the detected lipid mediators contained such major group terms as “Negative regulation of T cell receptor signaling pathway”, “Antigen processing and presentation exogenous lipid antigen via MHC class Ib”, “Regulation of immunoglobulin mediated immune response”. This cluster positively correlated (ρ = 0.62 to 0.75, p ≤ 6E-05) with DGLEA, AEA, DEA and NA-Gly. Negatively correlated (ρ = -06 to -0.78), p ≤ 0.0001) with 13-HODE and 15(16)-EpODE. Another cluster included “Alpha-beta T cell differentiation”, “Negative regulation of immune response”, “Regulation of RIG-1 signaling pathway”. Although correlation with eicosanoids was not statistically significant, this cluster had a similar heat-map pattern to the previous and adjacent cluster, and additionally, it had statistically significant GO terms. This cluster represents a relatively large network of multiple GO terms mostly representing various functions of cytotoxic T cells as well as Th cells in the immune response (Fig 4**, purple nodes**).

Interestingly, the correlation of these two clusters was positive with the acylethanolamides and negative with prostanoids, 13-HODE and epoxy linoleates (EpOMEs). These groups of oxylipins demonstrated reverse correlation. As it is demonstrated on the supplementary Fig S3, gene clusters that had positive correlation with prostanoids, 13-HODE and epoxy linoleates had negative correlation with acylethanolamides.

***Mediastinal lymph nodes –*** A total of 14 clusters of WGCNA transcriptomics modules (clusters of co-expressed genes) were identified in mediastinal lymph node tissue and 36 lipid mediators and ibuprofen were quantified by mass spectrometry (supplementary S4 Fig).

**Fig 5:**
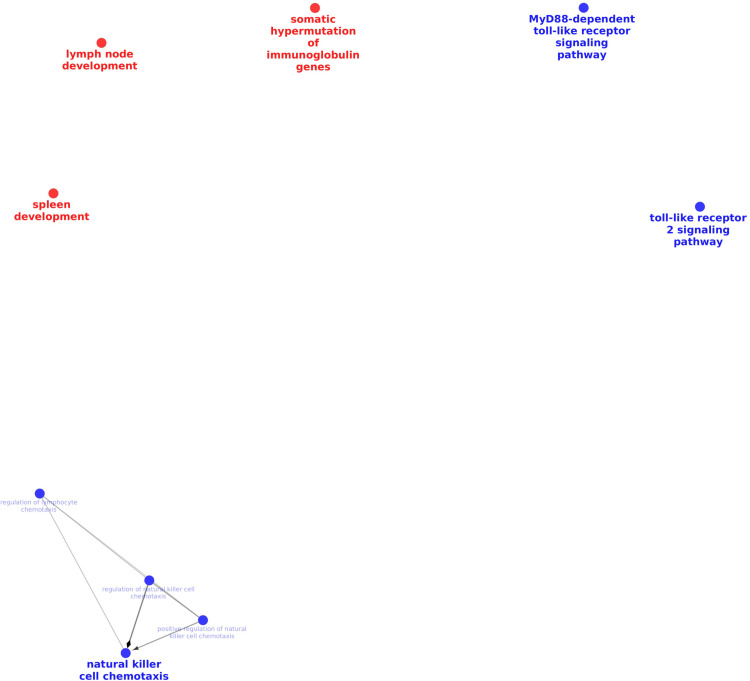
Result of network analysis of two clusters of co-expressed genes in mediastinal lymph node.

The cluster (**Fig 5, red nodes**) with major groups of co-expressed genes such as “Neutrophil activation” (statistically significant term), “Immunoglobulin heavy chain V-D-J recombination” (statistically significant term) and “Negative regulation of complement activation, classical pathway” positively correlated with DGLEA (ρ = 0.67, p = 7E-06), and negatively correlated with glyceryl linoleate 1-LG (ρ = -0.62, p = 6E-05).

The clusters were selected based on the fact that they have any correlations with some oxylipins.

Red - Neutrophil activation”, “Immunoglobulin heavy chain V-D-J recombination” and “Negative regulation of complement activation, classical pathway”; Blue - “Cell surface toll-like receptor signaling pathway”;

The next cluster (Fig 5**, blue nodes**) that had a statistically significant correlation spot was “Cell surface toll-like receptor signaling pathway” (Statistically significant and the only term in the entire cluster). It negatively correlated (ρ = -0.65, p = 2E-05) with 9,12,13-TriHOME.

There were no other correlations in mediastinal lymph nodes the reach the corrected p-critical value (i.e., p ≤0.0001) for the dataset.

General trends in the correlation heatmap were similar to those in bronchial lymph nodes, i.e., prostanoids and acylethanolamides appeared to have opposite correlation with most of the gene clusters, suggesting an antagonistic nature.

Immune function network analysis of these two clusters (**Fig 5**) showed no connections between them as well as no connections among groups within each cluster. Biological process network analysis of the same clusters did not demonstrate many connections between terms and their groups (Supplementary S5 Fig).

### 3.4. Only IL-13 and IL-1R2 expression levels correlated with ibuprofen in bronchoalveolar lavage samples

**Fig 6:**
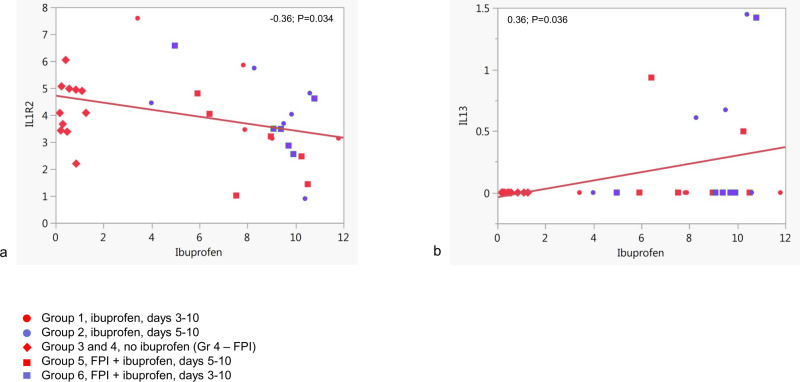
Correlation of cytokine gene expression and ibuprofen concentrations in bronchoalveolar lavage. Only statistically significant results are shown.

Normalized counts data for various cytokines, chemokines, their receptors and some immune function-related transcription factors were used to determine correlation with ibuprofen concentration (supplementary S8 File). No statistically significant correlation was found except negative correlation (ρ = -0.36; p = 0.034) with IL1R2 gene expression (Fig 6a) and positive correlation (ρ = 0.36; p = 0.036) with IL13 gene expression (Fig 6b).

### 3.5. Correlation of ibuprofen levels and expression of various immune function-related genes in bronchial lymph nodes

**Fig 7:**
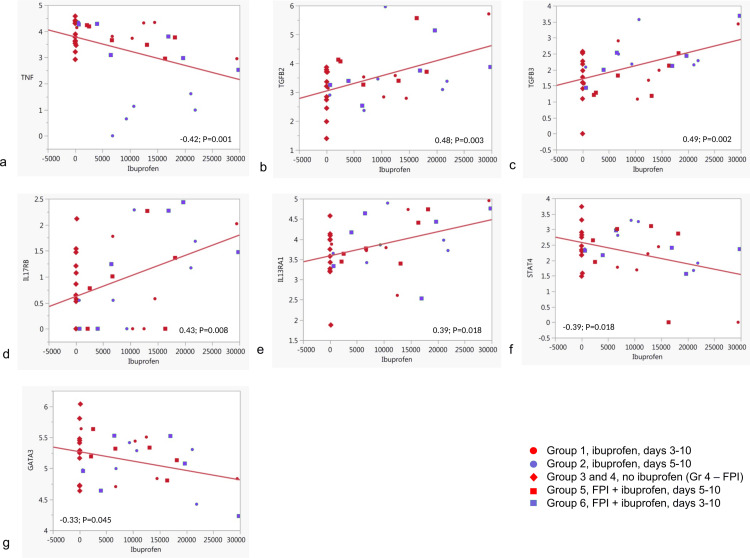
Correlation of cytokine-, cytokine receptor- and transcription factor gene expression and ibuprofen concentrations in bronchial lymph nodes. Only statistically significant results are shown.

Normalized counts data for various cytokines, chemokines, their receptors and some immune function-related transcription factors were used to determine correlation with ibuprofen concentration in BLN (supplementary S9 File). Tumor necrosis factor α (TNF-α) expression in bronchial lymph nodes showed negative correlation (ρ = -0.42; p = 0.001) with Ibuprofen levels measured in the same tissue (**Fig 7**). The lowest TNF expression levels were observed in the group 2 animals treated with only ibuprofen, starting on day 5. In other groups, levels of TNF expression were relatively higher than in group 2. Transforming growth factors beta 2 and 3 (TGFB2 and TGFB3) both demonstrated positive correlation (ρ = 0.48; p = 0.003 and ρ = 0.49; p = 0.002 respectively) with ibuprofen levels. Among cytokine receptors, IL17 receptor B (IL17RB) expression demonstrated positive correlation (ρ =0.43; p =0.008) with Ibuprofen concentration in BLN. IL-13 receptor alpha 1 (IL13RA1) expression was also positively correlated with ibuprofen in BLN (ρ = 0.39; p = 0.018). Several transcription factors also correlated with ibuprofen levels in BLN tissue. STAT4 expression showed negative correlation (ρ = -0.39; p = 0.018) with ibuprofen levels in BLN. Negative correlation (ρ = -0.33; p = 0.045) was also demonstrated in the expression of GATA3.

### 3.6. IL-17, IL-4 and interferon-γ gene expression in lymph nodes (qPCR analysis)

**Fig 8:**
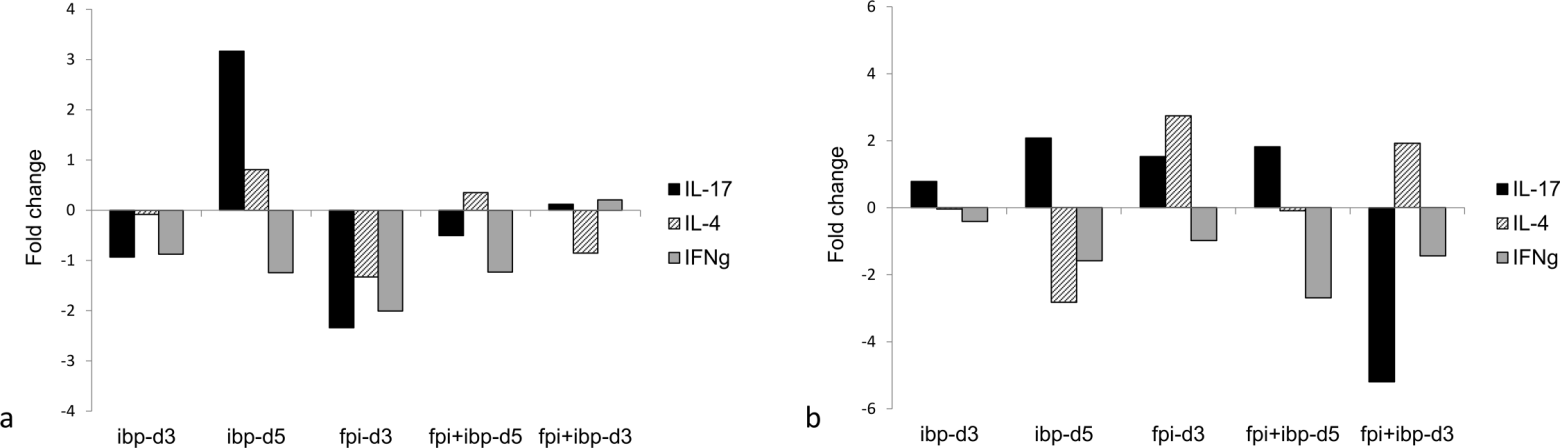
Results of the qPCR cell expression analysis of IL-17, IL-4 and interferon- **γ.** a – bronchial lymph nodes; b – mediastinal lymph nodes. No statistically significant differences in the expression of all cytokines were detected across treatment groups.

Despite high variability in results of gene expression measured by quantitative RT-PCR for these 3 cytokines, we observed certain trends in the average values of fold change among experimental groups. In bronchial lymph nodes from animals treated with ibuprofen only cytokine expression did not vary much from the placebo group as we can see in group ibp-d3 (Fig 8); or increased average production of IL-17 and IL-4 that was observed in group ibp-d5. The expression of all 3 cytokines was downregulated in group fpi-d3, where animals were treated with the FPI only. In groups fpi+ibp-d5 and fpi+ibp-d3 where both FPI and ibuprofen were administered, expression of all 3 cytokines did not go far beyond the placebo levels. In mediastinal lymph nodes IL-17 was upregulated in all groups except for the group fpi+ibp-d3 where it was downregulated. IL-4 demonstrated an opposite pattern compared to bronchial lymph nodes. Interferon gamma was mostly downregulated in all groups except for the group ibp-d3 where it was not different from the placebo group.

## 4. Discussion

The goal of this study was to evaluate the effect of the therapeutic interventions on the immune functions in bovine calves infected with BRSV. This study was designed with a dual purpose, not only to explore the effects of the treatment in calves but also to utilize them as a model for human respiratory syncytial virus infection. This dual-purpose approach was particularly significant in ensuring the findings could have broader implications for human medicine. Consequently, the selection of the NSAID was carefully aligned with this goal. Ibuprofen is not only relevant in veterinary medicine but is also widely used in human healthcare, including pediatric practice. This strategic choice ensures that the study’s results are more applicable and translatable to human clinical settings, enhancing the potential impact of the research.

We followed the dynamics of changes in frequencies of major groups of PBMC under experimental conditions and their ratios to the baseline levels as well as some more specific subsets of immune cells in the peripheral blood. These ratios helped determine relative changes in the population frequencies of different types of peripheral blood subsets by normalizing them to the corresponding baseline values. An increase or decrease in the experimental/baseline cell population ratios directly reflects numerical changes in these cell groups under experimental (treatment) conditions. Therefore, we can use these ratio changes to extrapolate relative changes in the cell populations under treatment conditions. An interesting finding was the similarity of some patterns of changes in animals from groups ibp-d5 and fpi+ibp-d5. These changes included statistically significant increases in Th cells and cytotoxic T cells in peripheral blood on day 8 after infection, followed by elevation of activated Th cells as well as memory Th cells. Groups ibp-d5 and fpi+ibp-d5 had a similar treatment regimen, as both groups were receiving ibuprofen starting from day 5. We can speculate that the later start of treatment was the factor, but then, results from placebo group and fpi-d3, where ibuprofen was not used, might be expected to be similar, but these groups had different results. Apparently, the start of the clonal expansion of virus-specific Th cells as a part of the adaptive immune response was connected to these changes. Day 7 to day 16 is an interval with the active growth of antigen specific clones of T cells and B cells [31]. The interval when we observed the increase of T cell major populations, and their activated and memory subsets also coincided with dramatic reduction in the viral loads in nasal swabs that occurred in these animals [22]. The appearance of high frequencies of activated Th cells and cytotoxic T cells could be associated with the abrupt reduction in viral loads observed.

Frequencies of T cells with the typical regulatory phenotype (CD4+CD25+FoxP3+) also significantly increased in animals of group 5 (fpi+ibp-d5) and 6 (fpi+ibp-d3). These two groups were receiving both FPI and Ibuprofen. The only difference in treatment regimen was the day when treatment was started for these 2 groups. Combination of these factors enhances regulatory immune factors which help to resolve the inflammatory process faster. This is an interesting observation as it was previously demonstrated that FoxP3+ T cells probably do not play a significant role in regulation in the bovine immune system and instead, the regulatory subset of γδ T cells plays a crucial role in the immune regulation in these animals [32]. Our result showed that FoxP3+ T cells may still contribute to the immune regulation, but additional study is required to test this hypothesis. γδ T cells, however, also tend to increase at the same period of time (day 8 - day 10 post infection) which may be also connected to their regulatory function, or other roles.

Integration of genomic and metabolomic data brought some interesting insights into connection between levels of eicosanoids and immune functions associated with clusters of co-expressed genes. BAL and regional lymph nodes (bronchial and mediastinal) were each measured on day 10 post-infection, and revealed both negative and positive correlation between some lipid mediators and immune functions. In the lavage samples it was clear that thromboxane B2 had positive correlation with the multiple adaptive immune functions strongly connected to each other, such as αβ T cell activation, antigen processing and presentation via MHC class II and cytokine production. Thromboxane B2 is a stable metabolite of its active precursor thromboxane A2 [33]. Thromboxane A2 is an active metabolite in the immune system and a regulator of T cell activation by promoting low avidity interactions between CD4 T cells and dendritic cells along with low avidity IgG response [34], contributing to the pathogenesis of RSV. Interestingly, a trihydroxy linoleic acid metabolite, 9,12,13-Trihyroxyoctadecenoic acid (9,12–13-TriHOME), had significant negative correlation with these immune functions. It has been previously reported that the 9R, 12R,13R-TriHOME, pinellic acid has an anti-inflammatory effect and is able to inhibit prostaglandin production such as prostaglandin D2 [35].

It was another cluster of co-expressed genes that had significant positive correlation with prostaglandin E2 levels in the BAL and lymph nodes. The most important part of the cluster was “negative regulation of viral-induced cytoplasmic pattern recognition receptor signaling pathway”. It also includes 2 leading GO groups responsible for the suppression of the toll-like receptor pathways and partly integrated into the Th cell activation network mentioned above. PGE_2_ was also responsible for the downregulation of the antimicrobial humoral immune response mediated by antimicrobial peptide. This suggests that increased PGE_2_ negatively affects toll-like receptor recognition and humoral immune response. Therefore NSAID therapy reduces this effect, and it may partially explain how ibuprofen contributed to the synergistic antiviral effect of its combination with FPI in lungs we previously described. The observed synergistic effect was characterized by a significant reduction in clinical scores, lung tissue damage, and viral infection (viral loads) in experimental groups where ibuprofen was administered in combination with FPI. In contrast, in groups where only ibuprofen was administered, viral loads were even higher than in the placebo group [21, 22], and the reduction in clinical scores and pathological changes in the lungs did not differ from the placebo [23]. FPI administered alone also did not affect clinical scores compared to the placebo and resulted in higher viral loads than the combined treatment with ibuprofen [22]. Additionally, in this study we have demonstrated the effect of ibuprofen on cytoplasmic protein production, including regulation of ribosomal machinery, suggesting positive effects on overall protein production.

Analysis of gene expression based on normalized counts and ibuprofen concentrations measured by mass-spectrometry in BAL showed ibuprofen levels to have negative correlation with IL-1 receptor type 2. This molecule is a decoy receptor for IL-1. By competing with IL-1 receptor type 1, the receptor type 2 helps to regulate IL-1 responsiveness of the cell [36]. The negative correlation between this receptor and ibuprofen levels may be explained by reduction of the inflammatory response characterized by either reduction of immune cells bearing the receptor in BAL or downregulation of the receptor expression to compensate for lack of IL-1 signaling by the negative feedback loop.

Ibuprofen correlated positively with IL-13 in BAL. Interestingly, IL-13 was expressed only in a few animals from groups 2, 5 and 6 (1–3 animals from each group), but no others. It is not clear whether it was or would be expressed in all animals during the course of the disease but reduced or delayed in most at the time of collection or if this was a result of individualized responses to NSAID treatment. It is possible that in these individual animals the response was tending towards an asthma-like scenario as we know that IL-13 is a key cytokine causing antibody isotype switching to the production of IgE [37] and it is well known that it is a typical scenario for BRSV pathogenesis [16, 17, 18]. Positive correlation with Ibuprofen may be explained by antagonistic relationship between Th1 and Th2 responses, and Ibuprofen as an anti-inflammatory agent was targeting mostly the Th1 response, which caused a balance shift to the Th2 phenotype, at least in some animals. These findings confirm and elucidate the immune mechanisms underlying our observation that combined therapy with an NSAID, and an antiviral significantly reduces clinical scores. The benefit is more pronounced when therapy is initiated three days post-infection rather than five days. Combined therapy resulted in lower clinical scores and improved respiratory rates [22].

Bronchial lymph nodes are the closest to the site of inflammation among all regional lymph nodes and are responsible for most of the immune reactions to lung infection. We also anticipated their response to the effect of NSAID to be the most prominent. BLN were sampled on day 10 when the disease was already in the active resolving phase, however WGCNA integrated with metabolomics data revealed a series of connections between oxylipin levels and multiple immune functions. For example, 13-Hydroxyoctadecadienoic acid (13-HODE, i.e., coriolic acid) positively correlated with the major immune functions, such as B cell differentiation, regulation of cytotoxic cells, Th1 cell cytokine production, myeloid cells and pattern recognition and leukocyte chemotaxis. Since all GO terms for these functions were not significant, we can only speculate that there is a direct or indirect connection between these GO terms and 13-HODE. Additionally, all the groups that we have in the network have relatively weak connections. These functional terms were all negatively correlated with ibuprofen levels suggesting that higher levels of 13-HODE are a result of infection. A similar effect of infection on elevated levels of 13-HODE was previously demonstrated with cytomegalovirus infection [38]. Additionally, 13-HODE as a product of lipoxygenase may be connected to the Th2 type of response as it was previously seen in asthma when lipoxygenases were activated by the asthmatic IL4/IL-13 type of immune response with elevated production of 12/15-lipoxygenase products such as 13-HODE in airway epithelial cells [39, 40]. This mechanism may have a similar pathophysiological role as BRSV infection has asthma-like cytokine milieu and mechanisms [15,16,17,18].

Some co-expressed gene clusters also had positives correlation with prostanoids (despite a p-value below significance (i.e., 0.02>p>0.003)) and negative correlations with endocannabinoids and endocannabinoid-like compounds, such as DGLEA), AEA, DEA and NA-Gly. These acylethanolamides and acylglycines may serve as antagonistic mediators to some oxylipins including prostanoids. In most cases, any gene cluster correlations with prostanoids were in reverse correlation relationships with these “endocannabinoids”. Additionally, our data suggests that critical immunoregulatory mechanisms, such as negative regulation of T cell receptor signaling pathway had strong positive correlations with higher levels of acylethanolamides and negatively correlated with many oxylipins, including prostanoids. It suggests that endocannabinoids may play a crucial role in the negative regulation of the adaptive immune response and resolving the inflammatory process, especially on the 10th day after infection when these mechanisms are expected to be prevalent. Our findings are concordant with the existing concept of the immunomodulating effect of endocannabinoids. It has been demonstrated that cannabinoid receptors CB1 and CB2 are expressed not only in neurons, but they are also abundant in cells of the immune system. As a result, endocannabinoids can suppress multiple functions of the immune system, such as innate responses, antigen presentation, inflammatory cytokine production, immune cell activation by inflammatory stimuli, chemotaxis and inflammatory cell migration [41].

Reverse correlations of some gene clusters with prostanoids and endocannabinoids may also be explained by the inhibiting effect of ibuprofen on cyclooxygenases and amidases that metabolize endocanabinoids. It has been previously demonstrated that ibuprofen inhibits metabolism of AEA with the same order of magnitude as required for inhibition of cyclooxygenase 2 [42].

Also, our observations suggest that such functions as neutrophil extravasation, lymphocyte differentiation and myeloid cell activation were positively connected to the group of oxylipins, such as the linoleate-derived 9,10- and 12,13-dihydroxyoctadecenoic acids (DiHOMEs). This cluster was negatively correlated with 15-hydroxyeicosatetraeneoic acid (HETE) and prostaglandins, weak positive correlation with endocannabinoids, and had the highest positive correlation with ibuprofen levels. Interestingly, DiHOMEs are produced by inflammatory myeloid cells, such as macrophages and neutrophils [43,44,45]. It has been also demonstrated that DiHOMEs are able to suppress the neutrophil respiratory burst [46]. Interestingly, according to our data, these oxylipins correlated negatively with prostanoids, but positively with ibuprofen levels, which suggests that they are directly or indirectly upregulated by ibuprofen and their role or the processes that lead to them may be crucial to relieve the oxidative stress which plays one of the most important roles in lung tissue damage caused by BRSV infection [24]. Histopathological analysis revealed that the greatest differences from placebo group were observed with dual therapy, particularly in the alveoli, septa, and bronchi in CDA. Moreover, consolidation was statistically significantly reduced only in calves receiving both ibuprofen and FPI on day 3 after inoculation. These findings suggest that the clinical benefits of combining FPI and NSAID therapy for BRSV are at least partially attributable to reduced lung tissue changes, especially when treatment begins three days post-infection. Ibuprofen alone mitigated lung alterations when started on day 3 but was less effective when initiated on day 5 [23].

Additional analysis of correlation between gene expression and ibuprofen levels in bronchial lymph nodes revealed statistically significant relationships among some cytokines, cytokine receptors and transcription factors responsible for immune functions. Among cytokines, as expected TNF had negative correlation with ibuprofen concentration, reflecting the anti-inflammatory effect of the NSAID. Conversely, TGF beta 2 and 3 demonstrated positive correlation with ibuprofen concentration as anti-inflammatory immunoregulatory cytokines. TGFβ superfamily cytokines are known to inhibit activation of T cells, differentiation and proliferation of major types of immune cells and many other aspects of the immune response [47] and, apparently, the anti-inflammatory effect of ibuprofen helped to shift the balance towards the anti-inflammatory environment in BLN. Among cytokine receptors, two of them showed positive correlation with ibuprofen levels. One of the receptors is IL-17 receptor B. The ligand for this receptor is IL-17B and it has been previously demonstrated that this cytokine performs an anti-inflammatory role [48]. The expression of another gene positively correlated with ibuprofen concentration was IL13RA1. It encodes IL13 receptor subunit α1. This result provides additional support for the thesis that ibuprofen caused shift in the immune regulation balance towards anti-inflammatory and immunosuppressive milieu, as it has been shown that IL13 receptor subunit α1 is a subunit critical for the signaling in both IL-13 and IL-4 receptors [49]. Among transcription factors gene expression, we showed that two genes negatively correlated with ibuprofen and one gene - positively. STAT4 is one of the negatively correlated transcription factors and it has been demonstrated that this factor is functionally important in the signaling pathways necessary for the immunity against intracellular pathogens and antiviral γ-interferon production [50, 51]. GATA3 is another transcription factor that was negatively correlated with ibuprofen in bronchial lymph nodes. This is a canonical Th2 transcription factor with multiple functions responsible for the maintenance of immune homeostasis [52]. Opposing effects of ibuprofen on these transcription factors can be explained by the fact that GATA3 limits Treg polarization toward an effector T cell phenotype and the production of effector cytokines [53]. This is why we have observed an antagonistic effect of ibuprofen on these transcription factors, where it may indirectly cause reduction in GATA3 expression, and increase in FoxP3, because of the reduction of limiting effect of GATA3. Additionally, GATA3 can indirectly inhibit viral infection [54]. This can be another explanation and a part of the mechanism by which ibuprofen treatment (without FPI) increased BRSV loads and virus shedding. Notably, combined therapy consistently outperformed ibuprofen alone, with additional benefits including reduced viral shedding when FPI and ibuprofen were administered together on day 3, in contrast to increased shedding observed with ibuprofen alone [22].

As expected, mediastinal lymph nodes demonstrated fewer interesting connections between co-expressed functional gene clusters and eicosanoid metabolites. Generally, patterns of negative versus positive correlations were similar to those in bronchial lymph nodes. The strongest positive correlation with prostanoids was demonstrated by functional gene groups responsible for regulation of leukocyte mediated cytotoxicity, T and B cell differentiation involved in the immune response, regulation of Th1 immune response and negative regulation of innate immunity. All these functions were negatively correlated to endocannabinoids.

## 5. Conclusions

1) Expansion of activated Th cells and cytotoxic T cells between days 6 and 8 helped to abruptly reduce viral loads, providing adaptive cellular immune response.
2) Combination of ibuprofen and FPI, regardless of treatment start day, caused an increase in T cell population with regulatory phenotype (CD4+CD25+FoxP3+) on day 10 post infection.
3) The population of total γδ T cells tended to expand on day 10 post infection with an increase in the group where ibuprofen treatment was started on day 3.
4) Ultimately, the synergistic effect of ibuprofen in combination with FPI may be explained by the positive effect of PGE_2_ suppression on cytoplasmic toll-like receptor recognition and humoral immune response mediated by antimicrobial peptides in lungs.
5) In both, lungs and mediastinal lymph nodes ibuprofen caused the shift in the cytokine, receptor and transcription factor environment towards anti-inflammatory (pro-Th2) status that is favorable to IL4/IL-13 responses, which needs to be taken into the account, especially when ibuprofen treatment is not combined with antiviral therapy.
6) Endocannabinoids in most cases showed reverse interactions with prostanoids and some other inflammation-associated oxylipins. Therefore, endocannabinoids may play a crucial role as natural regulators of inflammation, adaptive immune response and inflammatory resolution in response to viral infection.

## Supporting information

S1 Fig Correlations between metabolite and WGCNA transcriptomics modules in bronchoalveolar lavage. Each cell contains the Pearson correlation between indicated modules and metabolites, with the p-values shown in parentheses. Positive correlations are shown in red and negative correlations in blue, with the intensity of the color corresponding to the magnitude of the correlation. Due to the large number of tests conducted, only very small p-values (1e-4 or less) should be viewed as statistically significant.
(PDF)

S2 Fig Biological process network analysis in bronchoalveolar lavage. (PDF)

S3 Fig Correlations between metabolite and WGCNA transcriptomics modules in bronchial lymph nodes. Each cell contains the Pearson correlation between indicated modules and metabolites, with the p-values shown in parentheses. Positive correlations are shown in red and negative correlations in blue, with the intensity of the color corresponding to the magnitude of the correlation. Due to the large number of tests conducted, only very small p-values (1e-4 or less) should be viewed as statistically significant.
(PDF)

S4 Fig Correlations between metabolite and WGCNA transcriptomics modules in mediastinal lymph nodes. Each cell contains the Pearson correlation between indicated modules and metabolites, with the p-values shown in parentheses. Positive correlations are shown in red and negative correlations in blue, with the intensity of the color corresponding to the magnitude of the correlation. Due to the large number of tests conducted, only very small p-values (1e-4 or less) should be viewed as statistically significant.
(PDF)

S5 Fig Biological process network analysis in mediastinal lymph nodes. (PDF)

S1 File Representative examples of gating used in flow cytometry analysis. (PDF)

S2 File Quality control data for RNA used in transcriptomic analysis. (XLSX)

S3 File Parameters used in STAR analysis. (TXT)

S4 File Parameters used in HTStream preprocessing. (TXT)

S5 File Primers and probes used for qRT-PCR analysis of cytokines. (XLSX)

S6 File Raw and baseline-normalized data from flow cytometry. (XLSX)

S7 File Graphical presentation of baseline-normalized data for all cell populations across all time points and treatment groups. (PDF)

S8 File Expression of select immunologically significant genes in bronchoalveolar lavage analyzed using normalized counts from next-generation sequencing. (XLSX)

S9 File Expression of select immunologically significant genes in bronchial lymph nodes analyzed using normalized counts from next-generation sequencing. (XLSX)
