# Supplementary material for: Immune mechanisms affected by cyclooxygenase inhibition combined with antiviral treatment in calves infected with bovine respiratory syncytial virus
Source: PLoS One. 2025 Apr 22;20(4):e0321642. doi: 10.1371/journal.pone.0321642 (PMC12013931; doi:10.1371/journal.pone.0321642)
Supplement: S1 Fig — Each cell contains the Pearson correlation between indicated modules and metabolites, with the p-values shown in parentheses. Positive correlations are shown in red and negative correlations in blue, with the intensity of the color corresponding to the magnitude of the correlation. Due to the large number of tests conducted, only very small p-values (1e-4 or less) should be viewed as statistically significant. (PDF) [file pone.0321642.s001.pdf]

Module-Metabolite Correlations

Module	AA_cox1_TX_tbx2	AA_cox1_pg_ketoPGF1a	DGLA_cox2_pg_pge1	AA_cox2_PG_pge2	AA_cox2_pg_pgf2a	LTB4	AA_lox_triol_9_12_13_TriHOME	LA_vic_diol_seh_9_10_DiHOME	LA_r_oh_lox_13_HODE	LA_r_oh_lox_9_HODE	AA_r_oh_lox_12_HETE	AA_r_oh_lox_11_HETE	AA_r_oh_lox_5_HETE	LA_epox_cyp_12_13_EpOME	LA_epox_cyp_9_10_EpOME	aLA_epox_cyp_15_16_EpODE_pk1	aLA_epox_cyp_9_10_EpODE_pk2	AA_epox_cyp_14_15_EpETrE	OA_acyl_ea_pld_OEA	nsaid_treatment_Ibuprofen
Regulation of alpha-beta T cell activation 30.97%; Regulation of adaptive immune response 5.2%; Macrophage activation involved in immune response 4.26%	0.49 (2e-05)	-0.19 (0.1)	-0.061 (0.6)	-0.038 (0.8)	0.14 (0.3)	0.2 (0.1)	-0.58 (2e-07)	-0.25 (0.04)	0.17 (0.2)	-0.025 (0.8)	0.18 (0.1)	0.098 (0.4)	0.22 (0.08)	0.085 (0.5)	-0.077 (0.5)	-0.18 (0.2)	-0.36 (0.003)	0.01 (0.9)	0.085 (0.5)	-0.21 (0.09)
Natural killer cell proliferation 24.12%; B cell activation involved in immune response 13.83%; Lymphocyte activation involved in immune response 9.97%; Regulation of neutrophil migration 9.65%	0.37 (0.002)	-0.066 (0.6)	-0.062 (0.6)	-0.076 (0.5)	0.042 (0.7)	0.038 (0.8)	-0.34 (0.005)	-0.24 (0.04)	0.06 (0.6)	-0.065 (0.6)	0.043 (0.7)	0.091 (0.5)	0.27 (0.03)	0.072 (0.6)	0.039 (0.8)	0.025 (0.8)	-0.12 (0.3)	-0.12 (0.3)	0.035 (0.8)	-0.0014 (1)
**Regulation of immunoglobulin mediated immune response 60.71%**; Negative regulation of complement activation 17.86%*; Negative regulation of immune effector process 10.71%*; Positive regulation of B cell differentiation 10.71%***	-0.15 (0.2)	-0.05 (0.7)	0.23 (0.05)	0.13 (0.3)	0.059 (0.6)	-0.1 (0.4)	0.23 (0.06)	0.012 (0.9)	-0.078 (0.5)	0.096 (0.4)	-0.17 (0.2)	-0.23 (0.06)	-0.12 (0.3)	-0.15 (0.2)	-0.093 (0.5)	0.31 (0.01)	0.028 (0.8)	-0.2 (0.1)	0.098 (0.4)	0.11 (0.4)
**Ribosomal large subunit biogenesis 33.33%**; Ribosome assembly 33.33%**; Formation of cytoplasmic translation initiation complex 22.22%**; Cytoplasmic translation 11.11% ****	-0.0086 (0.9)	0.3 (0.01)	-0.39 (0.001)	-0.5 (1e-05)	-0.44 (2e-04)	0.078 (0.5)	0.3 (0.01)	0.2 (0.1)	-0.32 (0.007)	-0.23 (0.06)	0.17 (0.2)	0.26 (0.03)	0.29 (0.02)	-0.11 (0.4)	0.051 (0.7)	-0.07 (0.6)	0.25 (0.04)	-0.13 (0.3)	0.12 (0.3)	0.49 (2e-05)
Positive regulation of macrophage tolerance induction 50%; Isotype switching 25%; Positive regulation of neutrophil activation 16.67%; memory t cell differentiation 8.33 %	0.043 (0.7)	0.2 (0.1)	-0.12 (0.3)	-0.19 (0.1)	-0.15 (0.2)	-0.12 (0.3)	0.15 (0.2)	0.07 (0.6)	-0.14 (0.3)	0.018 (0.9)	0.015 (0.9)	0.17 (0.2)	0.13 (0.3)	-0.035 (0.8)	0.046 (0.7)	0.025 (0.8)	0.16 (0.2)	-0.16 (0.2)	0.011 (0.9)	0.32 (0.007)
**Negative regulation of isotype switching to IgE isotypes 60% *; Negative regulation of T cell proliferation 20% *; somatic hypermutation of immunoglobulin genes 20% ***	-0.13 (0.3)	0.04 (0.7)	0.16 (0.2)	-0.043 (0.7)	-0.094 (0.4)	-0.14 (0.3)	0.24 (0.05)	0.098 (0.4)	-0.17 (0.2)	-0.005 (1)	-0.0035 (1)	-0.076 (0.5)	-0.011 (0.9)	-0.093 (0.5)	-0.023 (0.9)	0.25 (0.04)	0.26 (0.03)	-0.25 (0.04)	0.091 (0.5)	0.18 (0.1)
Natural killer cell differentiation 28.74%, Macrophage activation involved in immune response 9.35%; Activation of innate immune response 5.61%	-0.0047 (1)	0.083 (0.5)	-0.022 (0.9)	-0.089 (0.5)	-0.094 (0.4)	-0.092 (0.5)	0.14 (0.3)	-0.047 (0.7)	-0.11 (0.4)	-0.05 (0.7)	-0.091 (0.5)	-0.0088 (0.9)	0.13 (0.3)	0.0031 (1)	0.11 (0.4)	0.19 (0.1)	0.19 (0.1)	-0.16 (0.2)	-0.0097 (0.9)	0.19 (0.1)
**Cell surface toll-like receptor signaling pathway 33.33% *, Regulation of pattern recognition receptor signaling pathway 33.33% **; Cytosolic pattern recognition receptor signaling pathway 33.33%***	0.065 (0.6)	0.2 (0.1)	-0.17 (0.2)	-0.17 (0.2)	-0.13 (0.3)	0.039 (0.8)	0.034 (0.8)	0.2 (0.1)	-0.023 (0.9)	-0.032 (0.8)	0.19 (0.1)	0.22 (0.08)	-0.023 (0.9)	-0.12 (0.3)	-0.15 (0.2)	-0.21 (0.08)	-0.13 (0.3)	0.024 (0.8)	0.072 (0.6)	0.14 (0.3)
**Positive regulation of antigen processing and presentation 47.62% **; Negative regulation of B cell activation 23.81%; Regulation of humoral immune response mediated by circulating immunoglobulin 14.29%**	0.13 (0.3)	-0.0078 (0.9)	0.12 (0.3)	0.096 (0.4)	0.078 (0.5)	0.0033 (1)	-0.15 (0.2)	-0.028 (0.8)	0.077 (0.5)	0.0083 (0.9)	0.085 (0.5)	-0.046 (0.7)	-0.089 (0.5)	-0.13 (0.3)	-0.25 (0.04)	0.028 (0.8)	-0.3 (0.01)	-0.034 (0.8)	0.2 (0.1)	-0.033 (0.8)
**Positive thymic T cell selection 50%; Positive regulation of MDA-5 signaling pathway 25%*; Isotype switching to IgG isotypes 25%**	-0.072 (0.6)	0.27 (0.03)	-0.082 (0.5)	-0.033 (0.8)	-0.093 (0.4)	-0.04 (0.7)	0.24 (0.05)	0.18 (0.1)	-0.13 (0.3)	0.029 (0.8)	0.036 (0.8)	0.022 (0.9)	-0.19 (0.1)	-0.21 (0.09)	-0.19 (0.1)	0.028 (0.8)	-0.016 (0.9)	0.012 (0.9)	0.057 (0.6)	0.081 (0.5)
**Mast cell degranulation 40%; Cell surface pattern recognition receptor signaling pathway 40% *; Regulation of cellular extravasation 20%***	-0.21 (0.08)	-0.15 (0.2)	0.28 (0.02)	0.27 (0.03)	0.18 (0.1)	-0.018 (0.9)	0.15 (0.2)	0.038 (0.8)	0.024 (0.8)	0.17 (0.2)	-0.14 (0.2)	-0.28 (0.02)	-0.28 (0.02)	-0.15 (0.2)	-0.17 (0.2)	0.18 (0.1)	-0.12 (0.3)	-0.035 (0.8)	0.092 (0.5)	-0.072 (0.6)
Regulation of pattern recognition receptor signaling pathway 66.67%; regulation of granulocyte chemotaxis 33.33%	-0.38 (0.002)	-0.13 (0.3)	0.0034 (1)	0.18 (0.1)	0.078 (0.5)	-0.0085 (0.9)	0.26 (0.03)	0.073 (0.6)	-0.0077 (1)	0.1 (0.4)	-0.27 (0.02)	-0.18 (0.2)	-0.14 (0.3)	0.12 (0.3)	0.24 (0.05)	0.096 (0.4)	0.28 (0.02)	0.093 (0.5)	-0.31 (0.01)	-0.12 (0.3)
**Positive regulation of megakaryocyte differentiation 50%**; Negative regulation of viral-induced cytoplasmic pattern recognition receptor signaling pathway 50%***	-0.34 (0.004)	-0.39 (9e-04)	0.27 (0.02)	0.6 (9e-08)	0.42 (4e-04)	-0.026 (0.8)	-0.0044 (1)	0.073 (0.6)	0.2 (0.1)	0.22 (0.07)	-0.32 (0.008)	-0.34 (0.005)	-0.38 (0.001)	0.048 (0.7)	0.037 (0.8)	0.19 (0.1)	-0.066 (0.6)	0.066 (0.6)	-0.38 (0.001)	-0.45 (1e-04)
Antimicrobial humoral immune response mediated by antimicrobial peptide 25%; Positive regulation of mast cell degranulation 25%; Regulation of CD4-positive, alpha beta T cell activation 12.5%; Positive regulation of activated T cell proliferation 12.5%	-0.14 (0.2)	0.11 (0.4)	-0.15 (0.2)	-0.46 (8e-05)	-0.33 (0.006)	0.087 (0.5)	0.35 (0.003)	0.22 (0.07)	-0.21 (0.08)	-0.096 (0.4)	0.16 (0.2)	0.2 (0.1)	0.19 (0.1)	-0.013 (0.9)	0.12 (0.3)	-0.24 (0.05)	0.34 (0.004)	-0.017 (0.9)	0.074 (0.5)	0.27 (0.02)
Mature B cell differentiation 21.03%; Negative regulation of leukocyte activation 17.76%; Mature B cell apoptotic process 12.62%	-0.3 (0.01)	-0.12 (0.3)	0.072 (0.6)	0.078 (0.5)	0.048 (0.7)	0.0056 (1)	0.23 (0.06)	0.18 (0.1)	0.066 (0.6)	0.15 (0.2)	-0.12 (0.3)	-0.068 (0.6)	-0.19 (0.1)	0.098 (0.4)	0.18 (0.1)	-0.1 (0.4)	0.19 (0.1)	0.14 (0.2)	-0.18 (0.1)	-0.12 (0.3)
**Response to type II interferon 50%*; Neutrophil chemotaxis 50%**	-0.18 (0.1)	-0.031 (0.8)	-0.051 (0.7)	0.031 (0.8)	0.013 (0.9)	0.097 (0.4)	0.12 (0.3)	0.16 (0.2)	0.014 (0.9)	0.093 (0.4)	0.024 (0.8)	-0.0071 (1)	-0.16 (0.2)	0.019 (0.9)	-0.00045 (1)	-0.19 (0.1)	0.051 (0.7)	0.17 (0.2)	-0.081 (0.5)	-0.13 (0.3)
Somatic diversification of immune receptors via germline recombination within a single locus 29.63%; Positive regulation of neutrophil migration 18.52%*; T cell receptor signaling pathway 14.81%	0.066 (0.6)	-0.084 (0.5)	-0.013 (0.9)	-0.16 (0.2)	-0.11 (0.4)	0.18 (0.1)	-0.0038 (1)	0.027 (0.8)	-0.13 (0.3)	-0.078 (0.5)	0.18 (0.1)	-0.0078 (0.9)	0.18 (0.1)	-0.089 (0.5)	-0.15 (0.2)	-0.046 (0.7)	0.039 (0.8)	-0.13 (0.3)	0.28 (0.02)	0.075 (0.5)

Color scale: 1, 0.5, 0, -0.5, -1
