# Supplementary figures and images for: Immune mechanisms affected by cyclooxygenase inhibition combined with antiviral treatment in calves infected with bovine respiratory syncytial virus
Source: PLoS One. 2025 Apr 22;20(4):e0321642. doi: 10.1371/journal.pone.0321642 (PMC12013931; doi:10.1371/journal.pone.0321642)

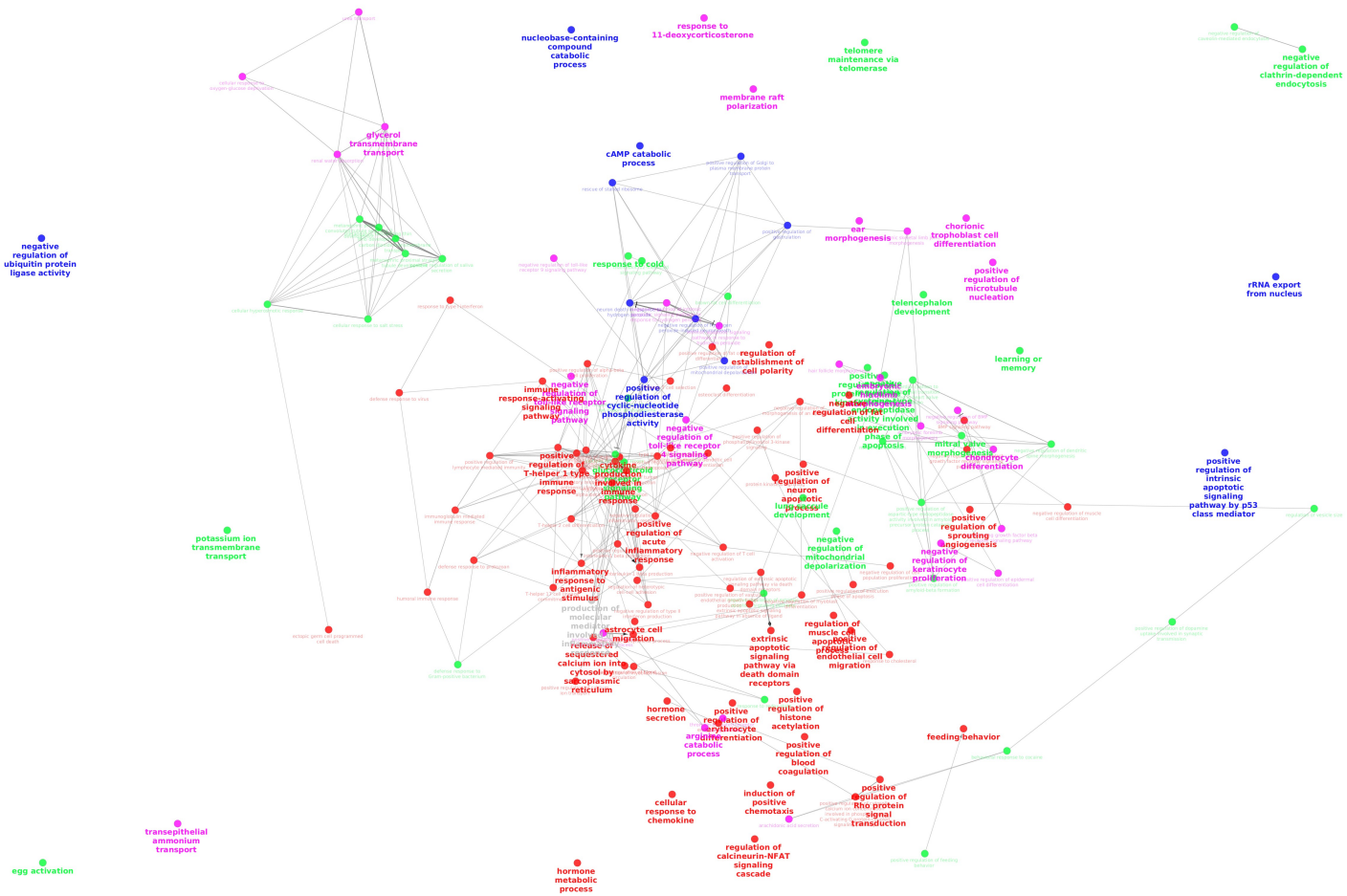

Supplement: S2 Fig — (PDF) [file pone.0321642.s002.pdf]

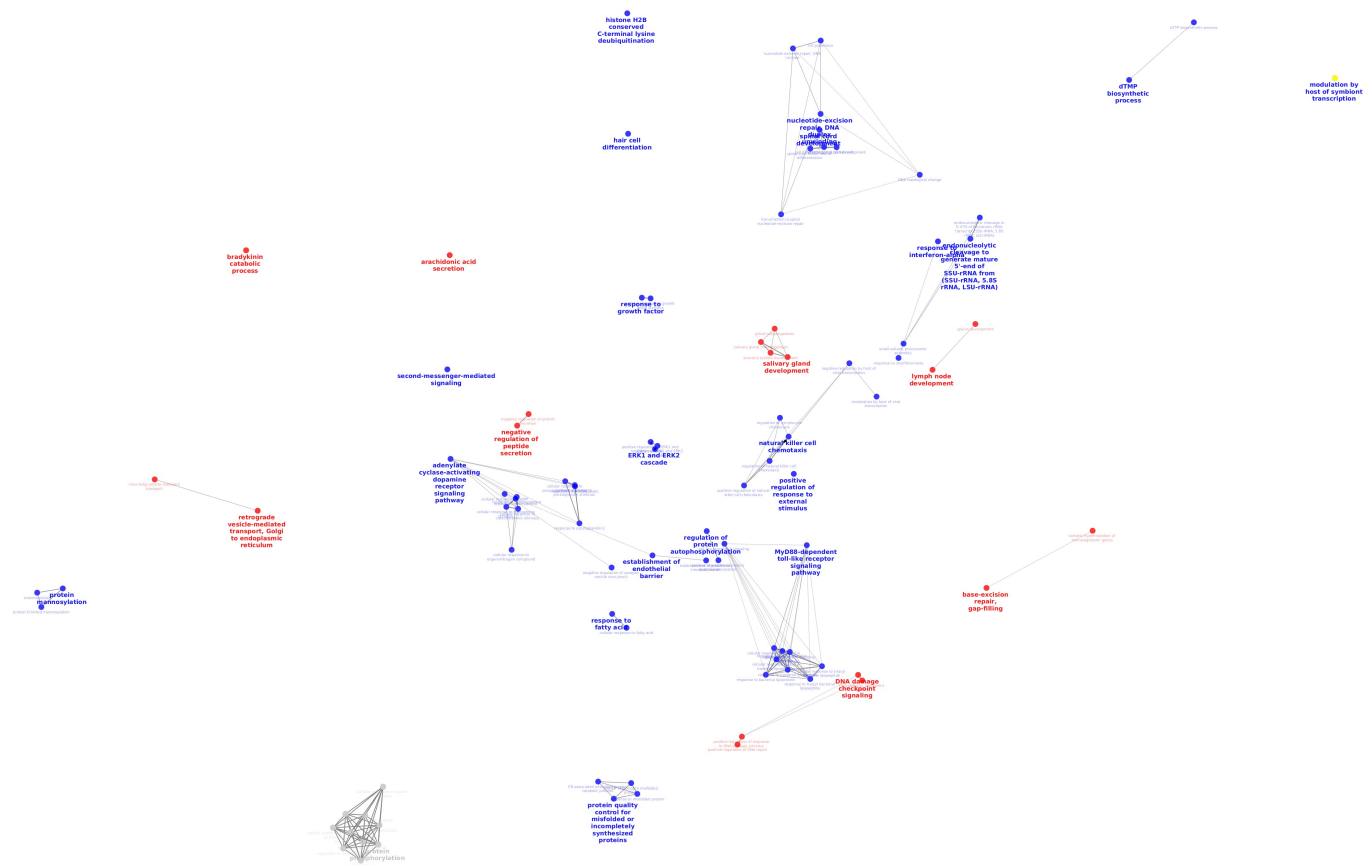

Supplement: S5 Fig — (PDF) [file pone.0321642.s005.pdf]

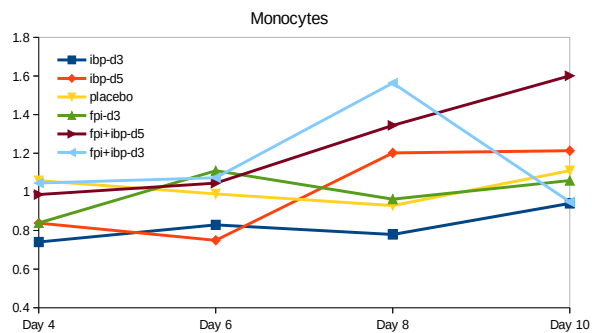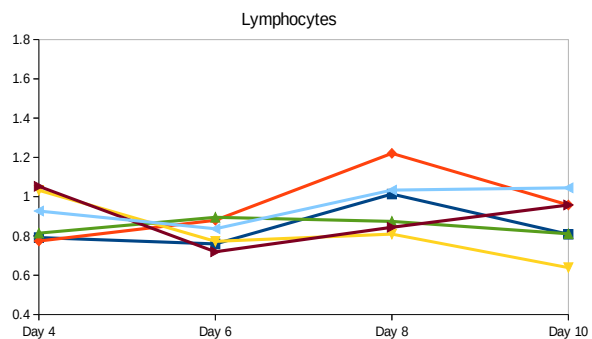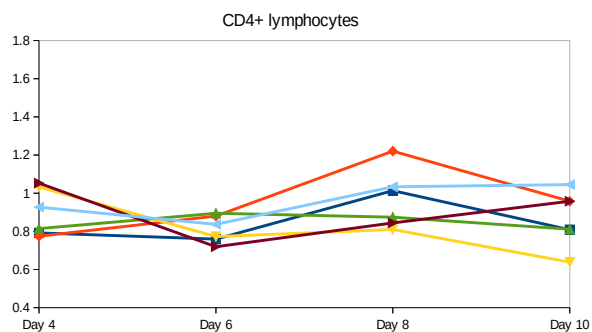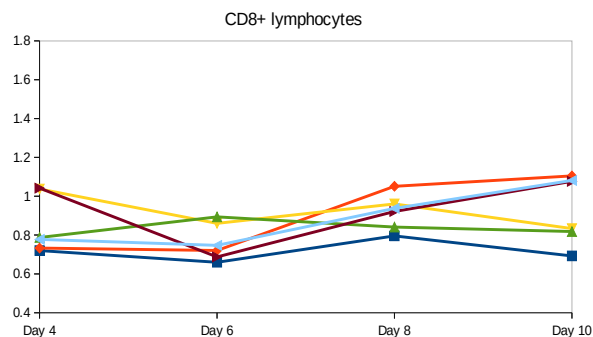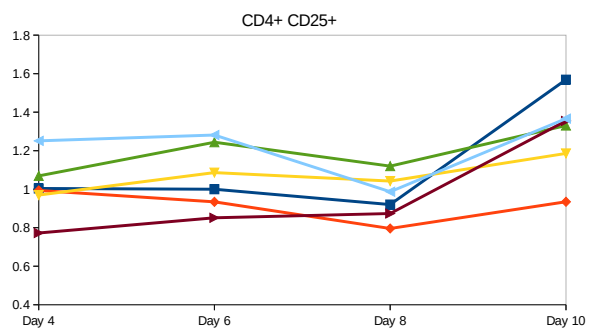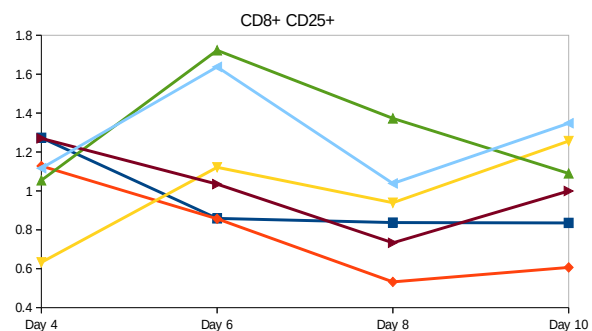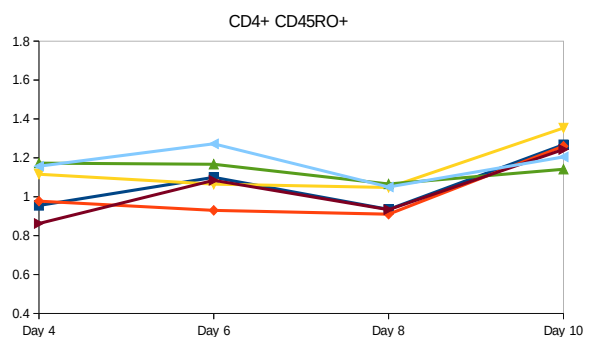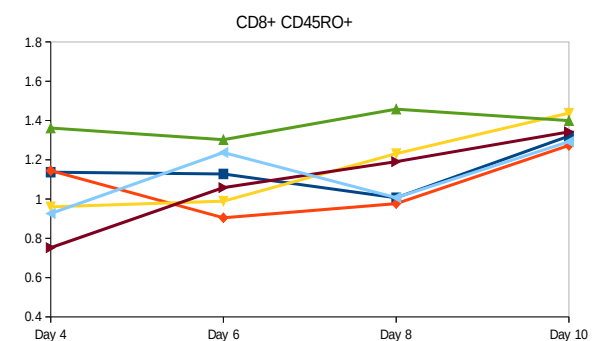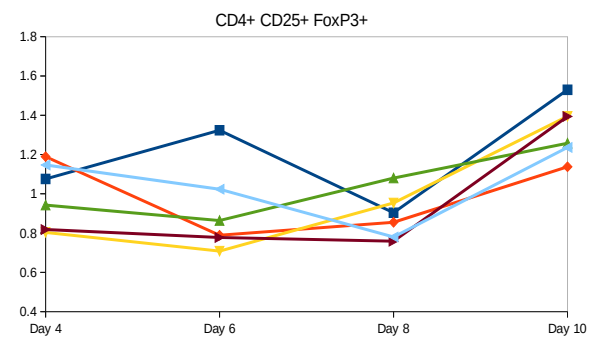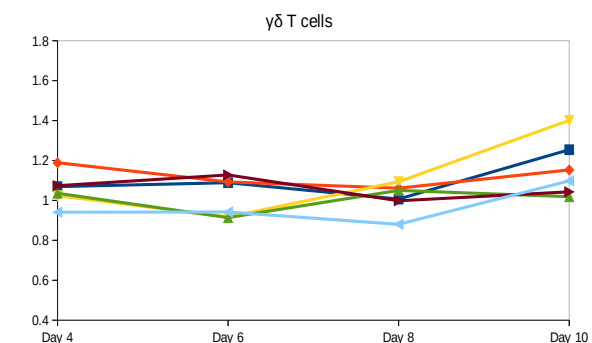

Supplement: S7 File — (PDF) [file pone.0321642.s012.pdf]
