# Supplementary material for: Immune mechanisms affected by cyclooxygenase inhibition combined with antiviral treatment in calves infected with bovine respiratory syncytial virus
Source: PLoS One. 2025 Apr 22;20(4):e0321642. doi: 10.1371/journal.pone.0321642 (PMC12013931; doi:10.1371/journal.pone.0321642)
Supplement: S3 Fig — Each cell contains the Pearson correlation between indicated modules and metabolites, with the p-values shown in parentheses. Positive correlations are shown in red and negative correlations in blue, with the intensity of the color corresponding to the magnitude of the correlation. Due to the large number of tests conducted, only very small p-values (1e-4 or less) should be viewed as statistically significant. (PDF) [file pone.0321642.s003.pdf]

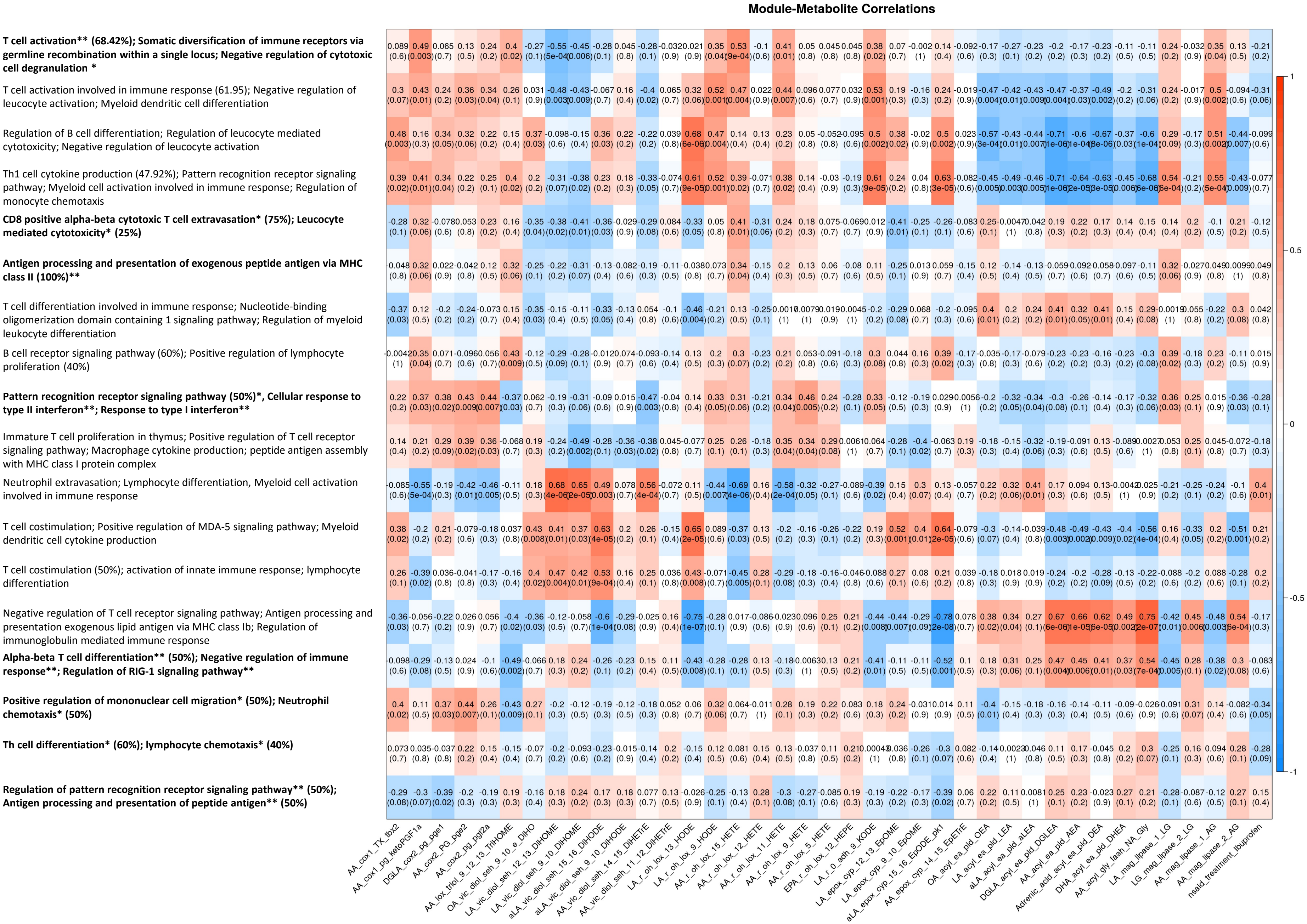

Module-Metabolite Correlations
T cell activation** (68.42%); Somatic diversification of immune receptors via germline recombination within a single locus; Negative regulation of cytotoxic cell degranulation *
T cell activation involved in immune response (61.95); Negative regulation of leucocyte activation; Myeloid dendritic cell differentiation
Regulation of B cell differentiation; Regulation of leucocyte mediated cytotoxicity; Negative regulation of leucocyte activation
Th1 cell cytokine production (47.92%); Pattern recognition receptor signaling pathway; Myeloid cell activation involved in immune response; Regulation of monocyte chemotaxis
CD8 positive alpha-beta cytotoxic T cell extravasation* (75%); Leucocyte mediated cytotoxicity* (25%)
Antigen processing and presentation of exogenous peptide antigen via MHC class II (100%)**
T cell differentiation involved in immune response; Nucleotide-binding oligomerization domain containing 1 signaling pathway; Regulation of myeloid leukocyte differentiation
B cell receptor signaling pathway (60%); Positive regulation of lymphocyte proliferation (40%)
Pattern recognition receptor signaling pathway (50%)*, Cellular response to type II interferon**; Response to type I interferon**
Immature T cell proliferation in thymus; Positive regulation of T cell receptor signaling pathway; Macrophage cytokine production; peptide antigen assembly with MHC class I protein complex
Neutrophil extravasation; Lymphocyte differentiation, Myeloid cell activation involved in immune response
T cell costimulation; Positive regulation of MDA-5 signaling pathway; Myeloid dendritic cell cytokine production
T cell costimulation (50%); activation of innate immune response; lymphocyte differentiation
Negative regulation of T cell receptor signaling pathway; Antigen processing and presentation exogenous lipid antigen via MHC class Ib; Regulation of immunoglobulin mediated immune response
Alpha-beta T cell differentiation** (50%); Negative regulation of immune response**; Regulation of RIG-1 signaling pathway**
Positive regulation of mononuclear cell migration* (50%); Neutrophil chemotaxis* (50%)
Th cell differentiation* (60%); lymphocyte chemotaxis* (40%)
Regulation of pattern recognition receptor signaling pathway** (50%); Antigen processing and presentation of peptide antigen** (50%)
AA_cox1_TX_tbx2
AA_cox1_pg_ketoPGF1a
DGLA_cox2_pg_pge1
AA_cox2_PG_pge2
AA_cox2_pg_pgf2a
AA_lox_triol_9_12_13_TriHOME
OA_vic_diol_seh_9_10_e_DiHO
LA_vic_diol_seh_12_13_DiHOME
LA_vic_diol_seh_9_10_DiHOME
aLA_vic_diol_seh_15_16_DiHODE
aLA_vic_diol_seh_9_10_DiHODE
AA_vic_diol_seh_14_15_DiHETrE
AA_vic_diol_seh_11_12_DiHETrE
LA_r_oh_lox_13_HODE
LA_r_oh_lox_9_HODE
AA_r_oh_lox_15_HETE
AA_r_oh_lox_12_HETE
AA_r_oh_lox_11_HETE
AA_r_oh_lox_9_HETE
AA_r_oh_lox_5_HETE
EPA_r_oh_lox_12_HEPE
LA_r_O_adh_9_KODE
LA_epox_cyp_12_13_EpOME
LA_epox_cyp_9_10_EpOME
aLA_epox_cyp_15_16_EpODE_pk1
AA_epox_cyp_14_15_EpETrE
OA_acyl_ea_pld_OEA
LA_acyl_ea_pld_LEA
aLA_acyl_ea_pld_aLEA
DGLA_acyl_ea_pld_DGLEA
AA_acyl_ea_pld_AEA
Adrenic_acid_acyl_ea_pld_DEA
DHA_acyl_ea_pld_DHEA
AA_acyl_gly_faah_NA_Gly
LA_mag_lipase_1_LG
LG_mag_lipase_2_LG
AA_mag_lipase_1_AG
AA_mag_lipase_2_AG
nsaid_treatment_ibuprofen
1
0.5
0
-0.5
-1
