# Supplementary material for: Immune mechanisms affected by cyclooxygenase inhibition combined with antiviral treatment in calves infected with bovine respiratory syncytial virus
Source: PLoS One. 2025 Apr 22;20(4):e0321642. doi: 10.1371/journal.pone.0321642 (PMC12013931; doi:10.1371/journal.pone.0321642)
Supplement: S4 Fig — Each cell contains the Pearson correlation between indicated modules and metabolites, with the p-values shown in parentheses. Positive correlations are shown in red and negative correlations in blue, with the intensity of the color corresponding to the magnitude of the correlation. Due to the large number of tests conducted, only very small p-values (1e-4 or less) should be viewed as statistically significant. (PDF) [file pone.0321642.s004.pdf]

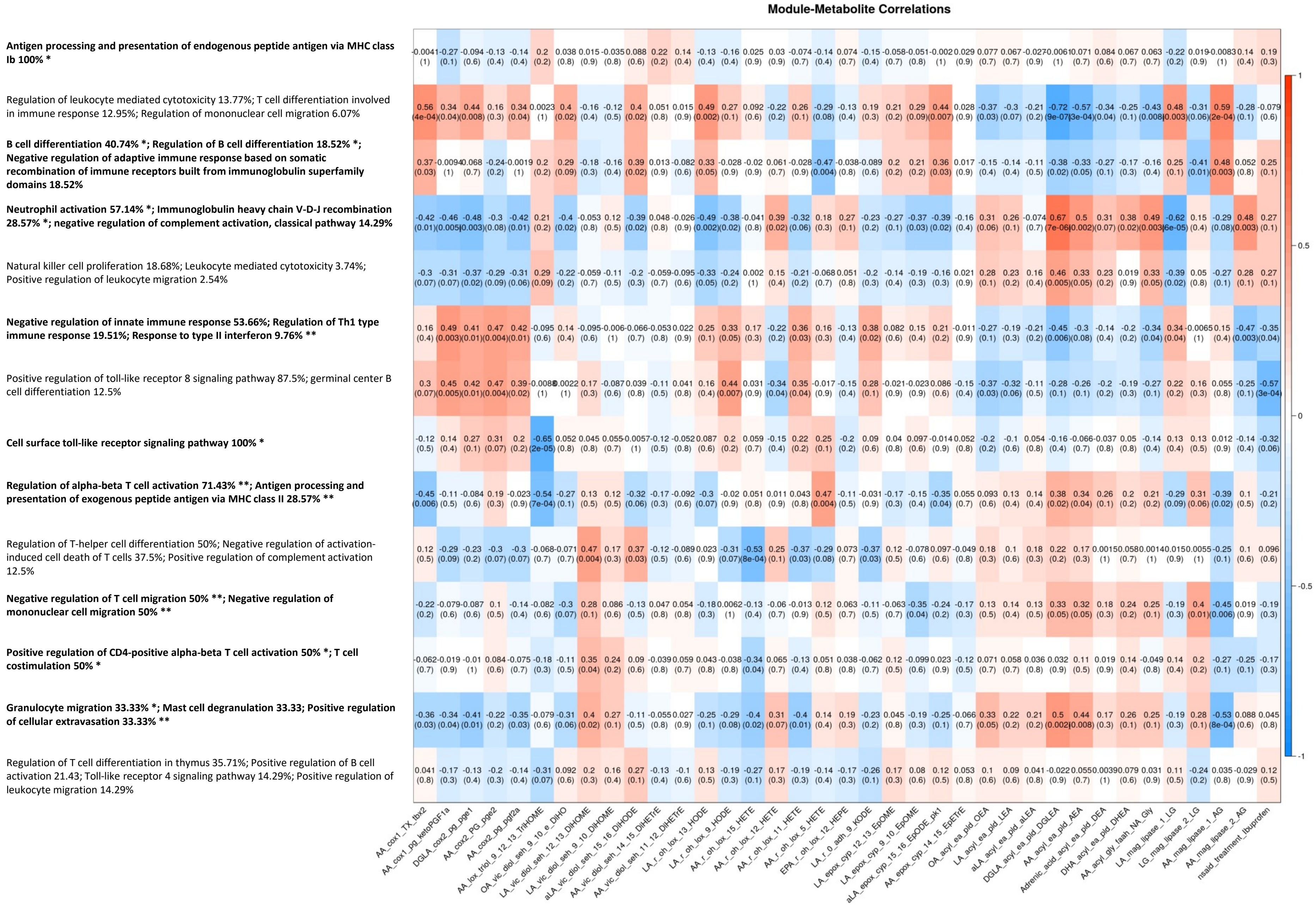

Module-Metabolite Correlations
Antigen processing and presentation of endogenous peptide antigen via MHC class Ib 100% *
Regulation of leukocyte mediated cytotoxicity 13.77%; T cell differentiation involved in immune response 12.95%; Regulation of mononuclear cell migration 6.07%
B cell differentiation 40.74% *; Regulation of B cell differentiation 18.52% *; Negative regulation of adaptive immune response based on somatic recombination of immune receptors built from immunoglobulin superfamily domains 18.52%
Neutrophil activation 57.14% *; Immunoglobulin heavy chain V-D-J recombination 28.57% *; negative regulation of complement activation, classical pathway 14.29%
Natural killer cell proliferation 18.68%; Leukocyte mediated cytotoxicity 3.74%; Positive regulation of leukocyte migration 2.54%
Negative regulation of innate immune response 53.66%; Regulation of Th1 type immune response 19.51%; Response to type II interferon 9.76% **
Positive regulation of toll-like receptor 8 signaling pathway 87.5%; germinal center B cell differentiation 12.5%
Cell surface toll-like receptor signaling pathway 100% *
Regulation of alpha-beta T cell activation 71.43% **; Antigen processing and presentation of exogenous peptide antigen via MHC class II 28.57% **
Regulation of T-helper cell differentiation 50%; Negative regulation of activation-induced cell death of T cells 37.5%; Positive regulation of complement activation 12.5%
Negative regulation of T cell migration 50% **; Negative regulation of mononuclear cell migration 50% **
Positive regulation of CD4-positive alpha-beta T cell activation 50% *; T cell costimulation 50% *
Granulocyte migration 33.33% *; Mast cell degranulation 33.33; Positive regulation of cellular extravasation 33.33% **
Regulation of T cell differentiation in thymus 35.71%; Positive regulation of B cell activation 21.43; Toll-like receptor 4 signaling pathway 14.29%; Positive regulation of leukocyte migration 14.29%
AA_cox1_TX_tbx2
AA_cox1_pg_ketoPGF1a
DGLA_cox2_pg_pge1
AA_cox2_PG_pge2
AA_cox2_pg_pgf2a
AA_lox_triol_9_12_13_TriHOME
OA_vic_diol_seh_9_10_e_DiHO
LA_vic_diol_seh_12_13_DiHOME
LA_vic_diol_seh_9_10_DiHOME
aLA_vic_diol_seh_15_16_DiHODE
AA_vic_diol_seh_14_15_DiHETrE
AA_vic_diol_seh_11_12_DiHETrE
LA_r_oh_lox_13_HODE
LA_r_oh_lox_9_HODE
AA_r_oh_lox_15_HETE
AA_r_oh_lox_12_HETE
AA_r_oh_lox_11_HETE
AA_r_oh_lox_5_HETE
EPA_r_oh_lox_12_HEPE
LA_r_0_adh_9_KODE
LA_epox_cyp_12_13_EpOME
LA_epox_cyp_9_10_EpOME
aLA_epox_cyp_15_16_EpODE_pk1
AA_epox_cyp_14_15_EpETrE
OA_acyl_ea_pld_OEA
LA_acyl_ea_pld_LEA
aLA_acyl_ea_pld_aLEA
DGLA_acyl_ea_pld_DGLEA
AA_acyl_ea_pld_AEA
Adrenic_acid_acyl_ea_pld_DEA
DHA_acyl_ea_pld_DHEA
AA_acyl_gly_faah_NA_Gly
LA_mag_lipase_1_LG
LG_mag_lipase_2_LG
AA_mag_lipase_1_AG
AA_mag_lipase_2_AG
nsaid_treatment_Ibuprofen
1
0.5
0
-0.5
-1
