# Supplementary material for: Immune mechanisms affected by cyclooxygenase inhibition combined with antiviral treatment in calves infected with bovine respiratory syncytial virus
Source: PLoS One. 2025 Apr 22;20(4):e0321642. doi: 10.1371/journal.pone.0321642 (PMC12013931; doi:10.1371/journal.pone.0321642)
Supplement: S1 File — (PDF) [file pone.0321642.s006.pdf]

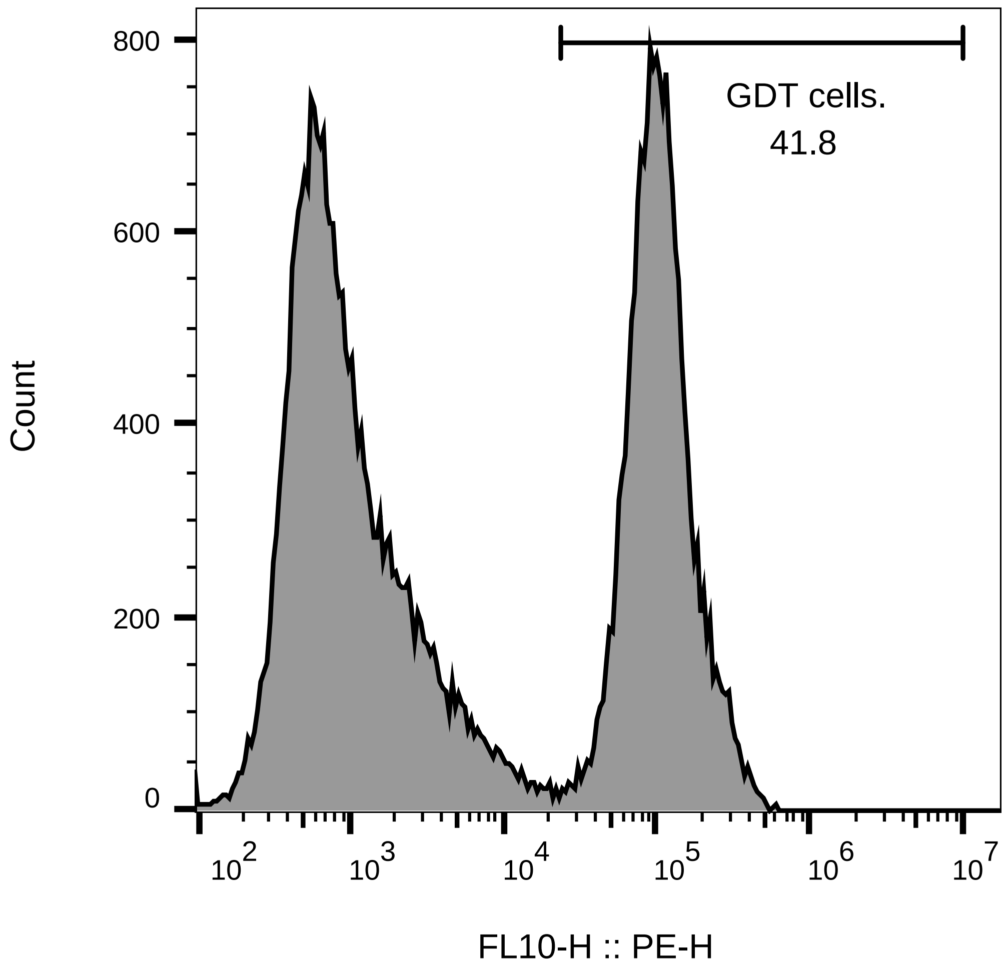

01-Well-A10.fcs
Lymphocytes
45336

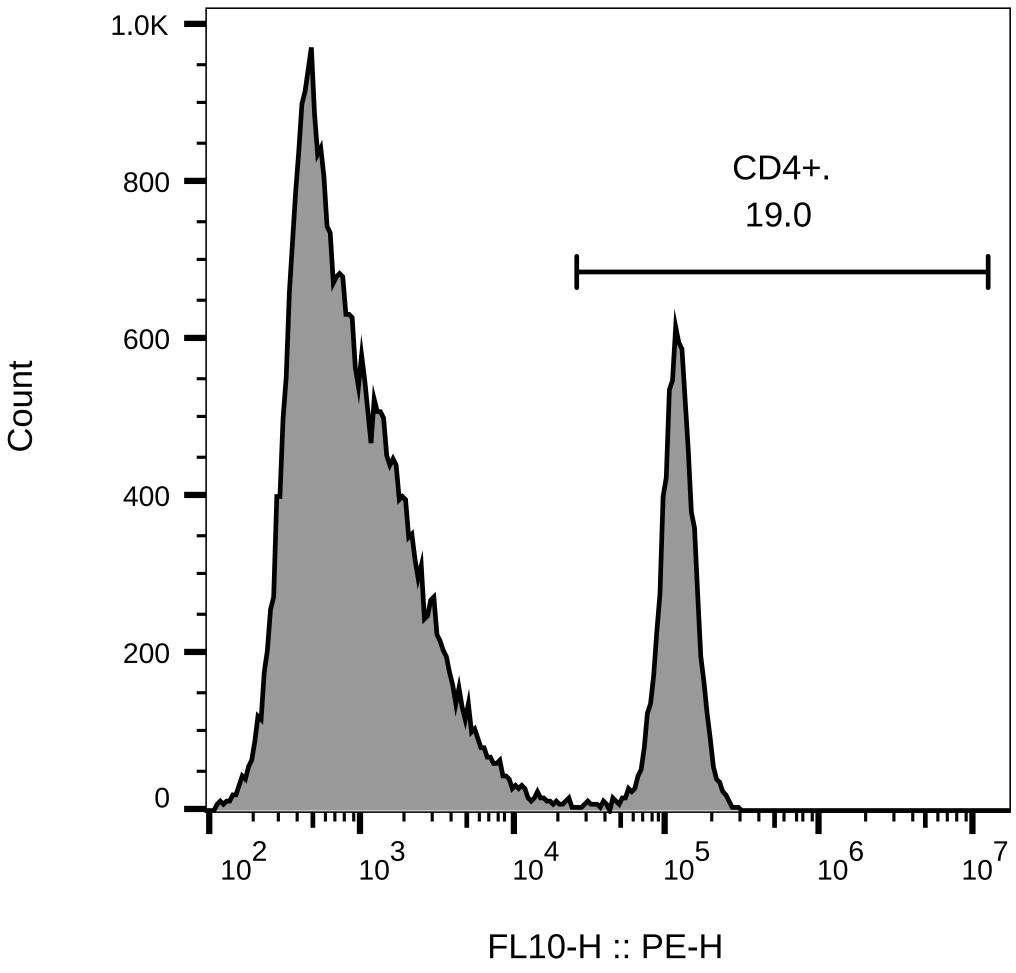

01-Well-C10.fcs
Lymphocytes
43709

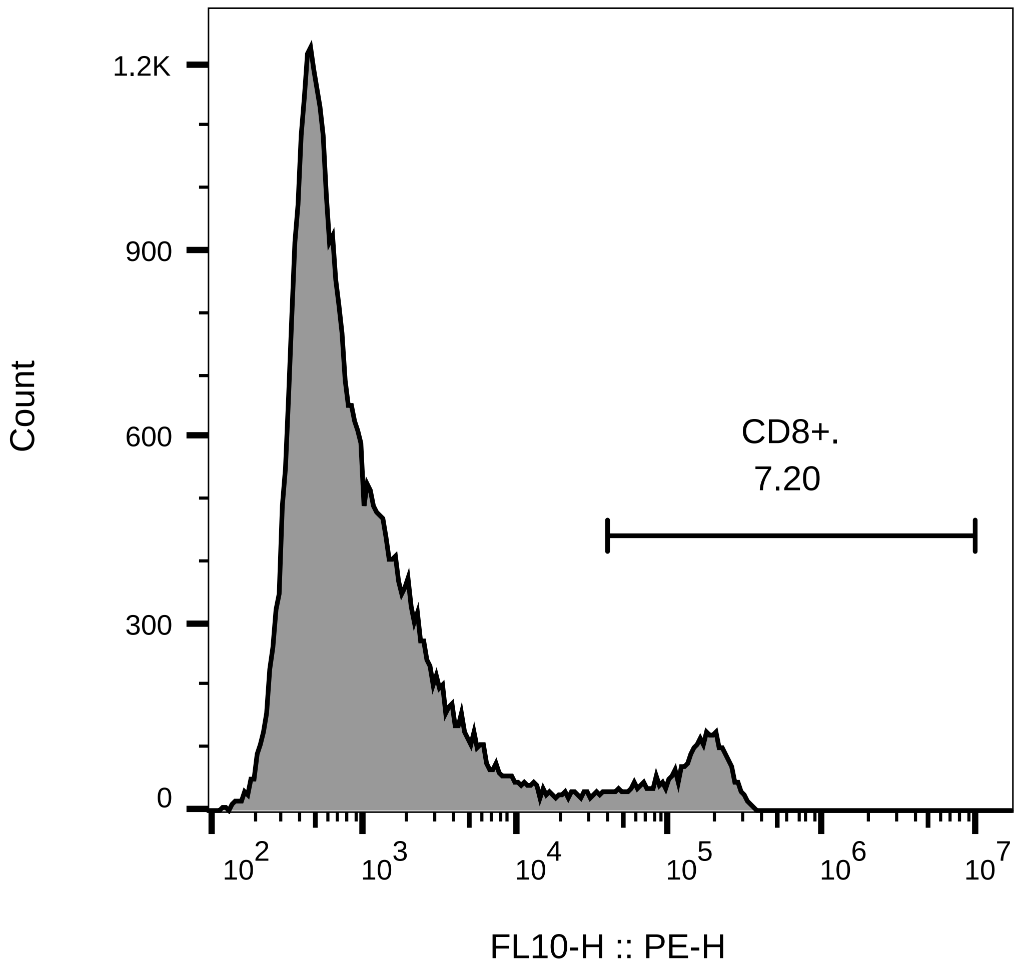

01-Well-B10.fcs
Lymphocytes
42235

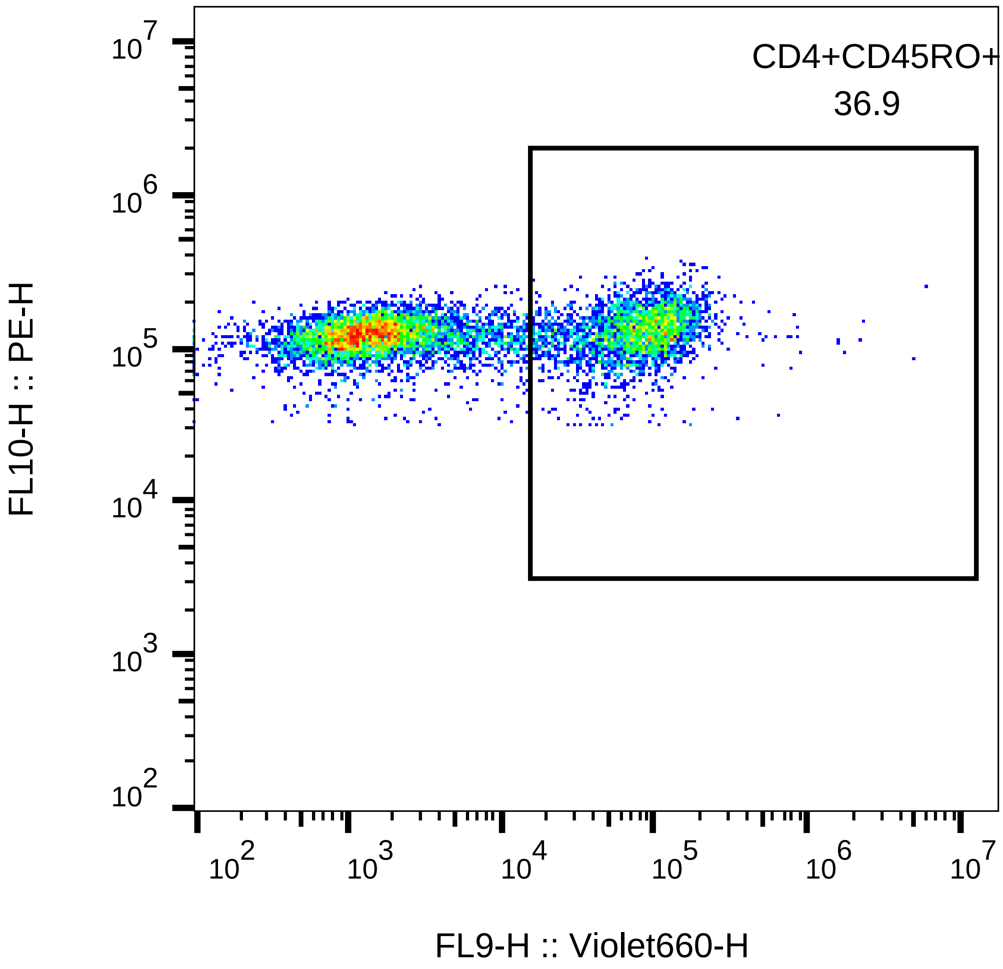

01-Well-C10.fcs
CD4+
8291

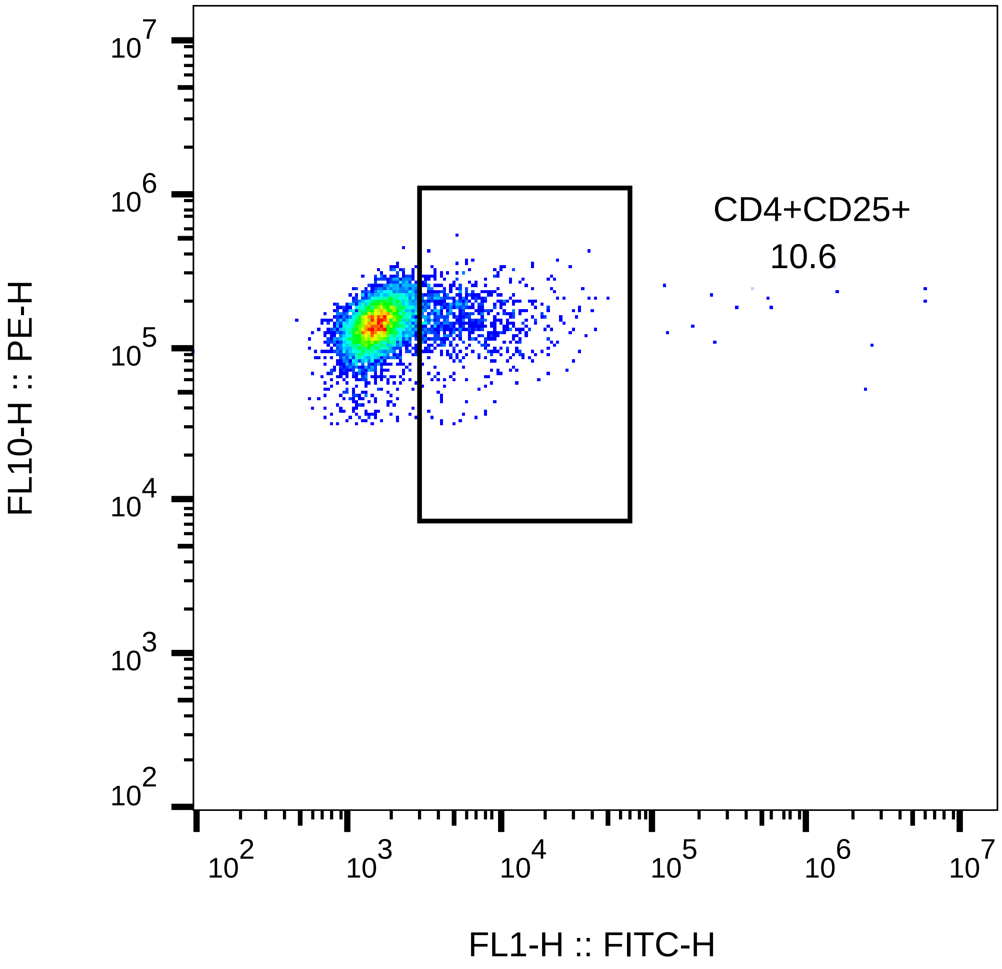

01-Well-C1.fcs
CD4+
8931

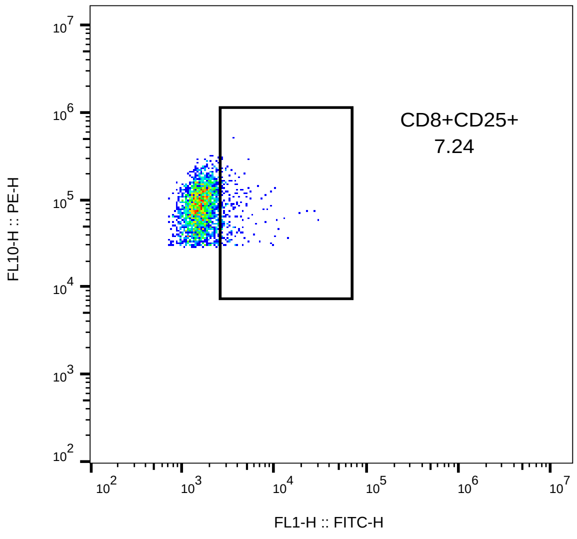

01-Well-F1.fcs
CD8+
2555

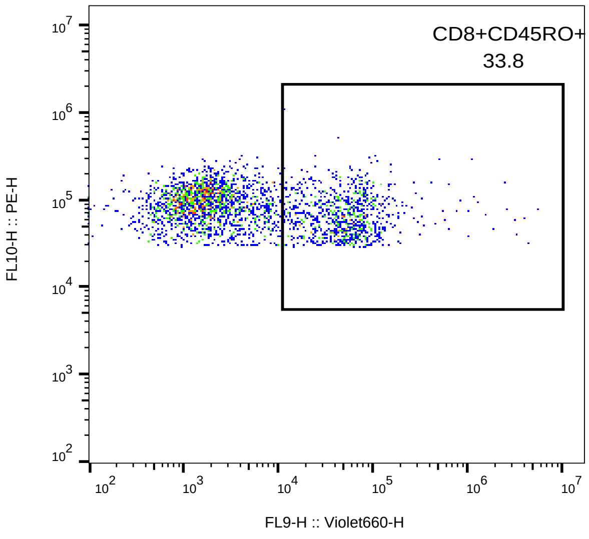

01-Well-F1.fcs
CD8+
2555

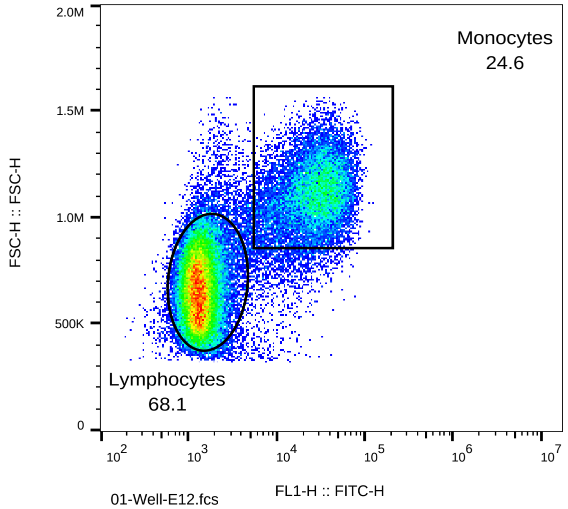

01-Well-E12.fcs
Live cells
59067

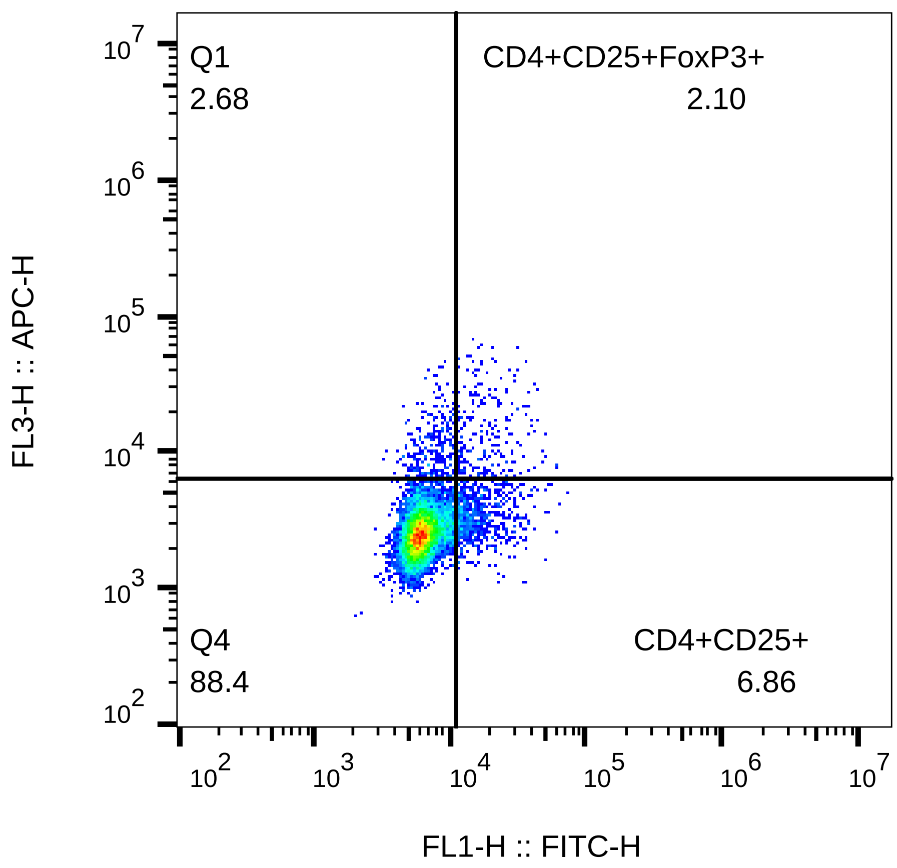

02-Well-B3.fcs
CD4+ cells
10929
